# TROPOMI CO and NO_2_ as Observational Constraints on the Sources of Air Pollution Inequalities in US Cities

**DOI:** 10.1021/acs.est.5c07455

**Published:** 2026-06-29

**Authors:** Xuehui Guo, Jon-Paul Mastrogiacomo, Debra Wunch, Isabella M. Dressel, Madeline A. Miles, Ziqi Gao, Andrew Gallego, Sally E. Pusede

**Affiliations:** 1 Department of Environmental Sciences, 2358 University of Virginia, Charlottesville, Virginia 22904, United States; 2 Department of Physics, 7938 University of Toronto, Toronto, Ontario M5S 1A7, Canada

**Keywords:** nitrogen dioxide, carbon monoxide, urban air pollution, environmental racism, environmental justice (EJ), TROPOMI

## Abstract

Carbon monoxide (CO) and nitrogen dioxide (NO_2_) are combustion pollutants that disproportionately harm communities of color in US cities. While distributive inequalities are established, we have lacked observational constraints on variability in air pollution mixtures and the sources that drive exposure disparities. We present the first application of the TROPOspheric Monitoring Instrument (TROPOMI) to describe census tract-scale CO inequalities and population-weighted NO_2_/CO enhancement ratios (ΔXNO_2_/ΔXCO) in six major US urbanized areas (UAs). Because CO has a long atmospheric lifetime, we develop an approach to describing CO inequalities using column-averaged dry-air mole fractions normalized by the daily urban variation (XCO_norm_). Annual daily mean inequalities in XCO_norm_ are significant in some UAs, e.g., Los Angeles, Phoenix, New York City, and Chicago, where they are 7–11% for Black, 7–15% for Latino, and 1–11% for Asian residents compared to non-Hispanic/Latino white residents; however, in Houston and Atlanta, inequalities in XCO_norm_ are near or indistinguishable from zero. We report direct satellite evidence of cumulative exposure burdens as CO and NO_2_ disparities are correlated across cities (*r* = 0.89). We compare inequalities on weekdays and weekends and with conditions that differently affect short- and long-lived, primary and secondary species, with evidence suggesting that CO inequalities are influenced not only by gasoline vehicles but also chemical production. TROPOMI ΔXNO_2_/ΔXCO indicate that combustion mixtures in primarily Black, Latino, and Asian neighborhoods are disproportionately influenced by heavy-duty diesel vehicles (HDDVs), even on weekends. To address exposure disparities, efforts should focus on differences in source composition not just magnitude. Spatial discrepancies between ΔXNO_2_/ΔXCO and the 2020 National Emissions Inventory (NEI20) suggest the NEI20, at least without downscaling, underpredicts the extent to which Black, Latino, and Asian neighborhoods are affected by high NO_2_/CO sources, e.g., HDDVs. Such errors in the NEI20 potentially direct attention and resources away from HDDV controls with environmental justice benefits.

## Graphical abstract

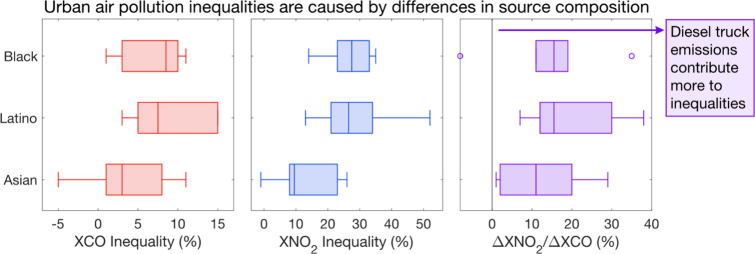

## 1 Introduction

Environmental racism affects neighborhood-level air quality in U.S. cities.^1–3^ Advances in satellite remote sensing^4–6^ and other techniques^3,7,8^ have improved quantitative descriptions of air pollution inequalities, especially for nitrogen dioxide (NO_2_) and particulate matter. However, there are few city-wide measurements of spatiotemporal distributions in pollutant mixtures or observational constraints on which emissions sources cause inequalities.^4,9^ Carbon monoxide (CO) and NO_2_ are produced by numerous urban combustion processes, and CO and NO_2_ enhancement ratios (ΔNO_2_/ΔCO) inform source patterns^10–12^ and, therefore, policy interventions.^12–15^ So far, satellite observations have only been used to study inequalities in pollutants individually, while cumulative exposures are often greater than those of single pollutants.^16^ Multipollutant inequalities based on concentrations from the same instrument or model provide context with which to interpret published research on disparities in single pollutants,^8^ e.g., NO_2_. Census tract-scale NO_2_ inequalities with race, ethnicity, and income have been described from space using the TROPOspheric Monitoring Instrument (TROPOMI), which also measures CO. TROPOMI CO inequalities and ΔNO_2_/ΔCO variability within cities have not yet been reported, with approaches developed for NO_2_ not readily applicable to CO. Neighborhood-level observations of CO, NO_2_, and ΔNO_2_/ΔCO would improve the interpretation of satellite NO_2_ columns as a combustion surrogate in health studies and knowledge of the sources that cause air pollution disparities, supporting decision-making and political accountability for environmental justice. Such analyses also challenge satellite observations analytically and push the bounds of their use in air quality research.

CO is both a primary and secondary pollutant that is harmful to our health,^17,18^ with an atmospheric lifetime of approximately one month. NO_2_ is emitted as NO_*x*_ (≡ NO + NO_2_), affects health,^19,20^ and has a chemical lifetime as short as a few hours in the summertime and up to a few days in winter.^21,22^ In U.S. cities, gasoline engines (on- and off-road) are often the largest anthropogenic source of primary CO, with emissions from industrial facilities and oil and gas operations potentially also important.^14,23^ Major NO_*x*_ emissions sources are heavy duty diesel vehicles (HDDVs), gasoline engines, electricity generation, and industrial activities.^23^ CO and NO_2_ spatial heterogeneities are affected by atmospheric dispersion, with decay length scales of a few hundred meters to 1–2 km away from sources.^24,25^ CO is also produced in the oxidation of gas-phase organic compounds, which can be important to total CO concentrations even in the urban atmospheric boundary layer (ABL).^14,26^ Secondary CO is more evenly distributed than primary CO, as chemical production is slower than time scales of dispersion. Liu et al.^8^ report the only U.S.-wide census tract-scale CO inequalities to our knowledge. With an empirical model, Liu et al.^8^ showed that population-weighted CO mixing ratios were higher for Black, Latino, and/or Asian population groups compared to the non-Hispanic/Latino white group. Differences between the most and least-exposed race-ethnicity groups were 16% for CO and 54% for NO_2_ across the U.S., with non-Hispanic/Latino white residents being the least-exposed to both pollutants. Liu et al.^8^ also estimated the largest absolute (−88%) and relative (−29%) changes in CO inequalities over 1990 to 2010 compared to other criteria pollutants, including NO_2_ (−54% and – 5%, respectively). These trends are consistent with emissions declines from gasoline-powered vehicles equipped with catalytic converters alongside smaller reductions in HDDV emissions,^27,28^ which are characterized by high NO_*x*_ relative to CO.^29^

Air pollutant enhancements ratios such as ΔNO_2_/ΔCO can be interpreted to identify emission sources and improve inventories.^12,13,30–37^ Different combustion sources emit CO and NO_*x*_ in distinct ratios according to fuel type, combustion efficiency, and control technology; as a result, analyses of trace species enhancement ratios can reveal differences in source contributions.^27^ Aircraft and ground-based studies using in situ measurements report substantial intraurban ΔNO_2_/ΔCO variability,^14,15,38,39^ and aircraft and satellite observations find large interurban ΔNO_2_/ΔCO differences from city-level differences in source mixtures.^12,13,27,28^ Inferring ΔNO_2_/ΔCO using TROPOMI and other satellite instruments is nontrivial because of differences in CO and NO_2_ chemical lifetimes and vertical measurement sensitivities that do not affect analyses of surface in situ datasets.^12,13^ TROPOMI has been shown to resolve intraurban NO_2_ variability and a major portion of the census tract-scale NO_2_ inequalities with race and ethnicity compared to those detected in airborne columns with pixels that subsample length scales of atmospheric dispersion.^4–6^ Applications of TROPOMI-derived CO inequalities and ΔNO_2_/ΔCO have the potential to provide actionable empirical constraints on which emissions sectors dominate the major sources of air pollution inequalities in U.S. cities, as well as inform evaluation of satellite-based ΔNO_2_/ΔCO generally.

We report the first space-based urban CO inequalities and corresponding TROPOMI census tract-scale NO_2_/CO enhancement ratios. Satellite observations are spatially comprehensive, at least when skies are cloud free, and can be used to describe intraurban spatiotemporal variability unmeasured at the surface. Because this is a new application of TROPOMI, we present our considerations, approach, and uncertainties in detail for a subset of six major U.S. Census-designated urbanized areas (UAs): Los Angeles–Riverside, California; Houston, Texas; Phoenix, Arizona; Chicago, Illinois; New York City–Newark, New York–New Jersey; and Atlanta, Georgia. We entwine method development and application, as it is through the application of the observations and comparisons between various inequality metrics that we produce evidence justifying our approach and results. Because we lack the fine spatial-scale airborne remote sensing measurements that have been foundational in establishing the use of TROPOMI to describe census tract-scale NO_2_ inequalities,^5,6,40^ we evaluate CO inequalities in the context of well-characterized NO_2_ columns, comparing CO inequalities on weekdays and weekends and evaluating CO and NO_2_ inequalities with environmental conditions that differently affect long- and short-lived and primary and/or secondary species. We compute census tract-scale TROPOMI NO_2_/CO enhancement ratios, producing new observational evidence that distributive inequalities in combustion pollution reflect disproportionate exposures for Black, Latino, and Asian neighborhoods to air mixtures influenced by sources with high NO_2_/CO, specifically HDDVs. Finally, we compare TROPOMI ΔNO_2_/ΔCO and the U.S. Environmental Protection Agency (EPA) National Emissions Inventory (NEI), a tool that guides multilevel air quality decision-making when used together with an air quality model.

## 2 Measurements and Methods

### 2.1 TROPOMI

TROPOMI measures CO and NO_2_ from a sun-synchronous orbit aboard the European Agency Copernicus Sentinel-5 Precursor (S-5P) satellite. The S-5P satellite has an equatorial crossing time of ∼1:30 pm local time (LT) and daily global coverage. CO full vertical column densities (VCDs), typically known as total vertical column densities, are inferred from observations in the 2315–2338 nm spectral region and a retrieval that also produces column averaging kernels and associated errors under clear-sky conditions.^41^ Our analysis includes CO pixels with quality assurance values of at least 0.7 for clear-sky and midlevel cloud conditions.^42^ We note that CO is elevated over the coastlines of New York City–Newark (Figure S1), which becomes less prominent when we remove CO pixels over water and could be an artifact of the retrieval algorithm over water surfaces.^43,44^ We follow the literature convention of retaining the CO pixels over water, which have a negligible effect on inequalities in XCO_norm_ (defined below) (Table S1). NO_2_ tropospheric vertical column densities (TVCDs) are derived by fitting the 405–465 nm spectral region, with the retrieval based on an updated version of the Dutch OMI (Ozone Monitoring Instrument) NO_2_ (DOMINO) algorithm and Quality Assurance for Essential Climate Variables project.^45–49^ We use Level 2 NO_2_ files and apply a quality assurance value filter of 0.75 (clear-sky conditions), as recommended.^50^ Census tract-scale NO_2_ inequalities based on TROPOMI NO_2_ TVCDs have been evaluated in detail,^4–6,51^ which is summarized in SI Appendix 1. From 1 May 2018 to 5 August 2019, the TROPOMI nadir spatial resolution was 7 km × 7 km for CO and 7 km × 3.5 km for NO_2_ (along track × cross track). From 6 August 2019, the nadir spatial resolution improved to 5.5 km × 7 km and 5.5 km × 3.5 km for CO and NO_2_, respectively. We use CO averaging kernels as is and NO_2_ averaging kernels adjusted by an airmass factor.^50^

CO VCDs and NO_2_ TVCDs are converted to column-averaged dry-air mole fractions, XCO and XNO_2_ with units of ppb, using the retrieved full (total) water column and surface pressure.^52^ Compared to vertical column densities, XCO and XNO_2_ are less sensitive to water vapor and pressure variations but are subject to uncertainties in surface pressure levels. We test the sensitivity of census tract-scale inequalities in XCO_norm_ to the pressure product, computing inequalities using pressure grids interpolated from the 1° × 1° European Centre for Medium-Range Weather Forecasts (ECMWF) reanalysis and as modeled for the TROPOMI NO_2_ retrieval. Census tract-scale inequalities in XCO_norm_ are not statistically significantly different between pressure products (Table S2), so we use the modeled TROPOMI product for both XCO and XNO_2_. We pair CO and NO_2_ pixels by assigning CO pixels to co-located NO_2_ pixels when they encompass the NO_2_ pixel center point. Note: XCO and XNO_2_ are column measurements and should not be confused with surface-level mixing ratios even though the units are the same.

### 2.2 Inequalities

For a schematic of our workflow, see Figure S2. Briefly, (1) paired TROPOMI CO and NO_2_ pixels are averaged daily using an approximated area-weighting within 2020 census tract polygons. Census tracts contain 2500–8000 people with boundaries that often follow visible landscape features. (2) Daily census tract-averaged XCO, CO VCDs, XNO_2_, and NO_2_ TVCDs are population weighted over the UA (eq S1)^7^ for people identifying in the census as non-Hispanic/Latino Black and African American (Black), Hispanic and Latino of any race (Latino), non-Hispanic/Latino Asian (Asian), and non-Hispanic/Latino white. (3) Absolute inequalities are calculated as the daily difference in mean population-weighted measurements between Black, Latino, or Asian residents and non-Hispanic/Latino white residents in the subset of tracts where the population percentage of that group (Black, Latino, Asian, or non-Hispanic/Latino white residents) is equal to or greater than the UA mean. Absolute inequalities are the difference in the averages between tracts, rather than the average difference within tracts. (4a) Relative inequalities (XCO_norm_ and XNO_2,norm_ inequalities) are computed as absolute inequalities divided by the daily UA interquintile range in census tract-scale CO and NO_2_ (eq S2). We use the interquintile range as opposed to the interquartile range (IQR) to fully reflect the range of variability within UAs; we use the interquintile range as opposed to the interdecile range to reduce noise at the tails. For reference, see a sample timeseries of XCO with the corresponding UA-level eightieth and twentieth percentiles and map of the frequency of observations UA-wide below the twentieth percentile and above the eightieth percentile (Figure S3). (4b) For comparison, we compute relative inequalities in columns (Table 2) equal to absolute inequalities divided by the mean of the population-weighted values for the two groups, which is always the differences in means for Black, Latino, or Asian residents and non-Hispanic/Latino white residents as defined above (eq S3). Uncertainties in inequalities are computed as the standard errors of the mean of daily inequalities. Inequalities based on the subset of tracts with larger populations for each group relative to the UA on average are likely larger than based on all tracts.^40,53^ While there is no standardized metric for inequalities, many states identify what may be referred to as “disadvantaged communities,” or using other terms, for consideration in regulatory, planning, and budgetary decision-making for environmental justice. These metrics typically focus on some subset of census tracts with larger populations of minoritized and marginalized residents and/or higher environmental burdens.

Mean daily TROPOMI census tract-scale inequality estimates are potentially affected by both (a) the number of census tracts within the UA with observations and (b) the size of TROPOMI pixels.^6^ (a) We test this sensitivity (SI Appendix 2), deriving daily coverage requirements for daily mean inequalities in XCO_norm_ and XNO_2,norm_ annually and seasonally of typically 10% (Tables S3 and S4) that are applied throughout. (b) Because census tracts are optimized for population, they are unlike TROPOMI pixels, which have a set of defined, although variable, spatial areas. As a result, there is wide variability in the spatial overlap of census tracts and XCO and XNO_2_ observations. Variability in TROPOMI pixels can be used to test the pixel size dependence of XCO_norm_ and XNO_2,norm_ inequalities.^6^ We find annual mean daily XCO_norm_ and XNO_2,norm_ inequalities to be insensitive to TROPOMI pixel size in each UA (Table S5). We combine the UA boundaries of Los Angeles–Long Beach–Anaheim and Riverside–San Bernardino, which we call Los Angeles–Riverside, and we refer to the Phoenix–Mesa UA as Phoenix. We report inequalities over June 2018–February 2023, excluding March 2020–February 2021 because of activity changes related to the COVID-19 pandemic.^54–56^ Key CO and NO_2_ metrics are defined in Table 1.

**Table 1:** Definitions of the Various CO and NO_2_ Metrics Used in This Work

**Abbreviation**	**Definition**	**Equation**
CO VCDs	CO full vertical column densities	–
NO_2_ TVCDs	NO_2_ tropospheric vertical column densities	–
XCO	CO column-averaged dry-air mole fractions	–
XNO_2_	NO_2_ column-averaged dry-air mole fractions	–
CO VCD_norm_	CO column density inequalities normalized by the daily UA variation (interquintile range)	eq S2
NO_2_ TVCD_norm_	NO_2_ tropospheric column density inequalities normalized by the daily UA variation (interquintile range)	eq S2
XCO_norm_	CO dry-air mole fraction inequalities normalized by the daily UA variation (interquintile range)	eq S2
XNO_2,norm_	NO_2_ dry-air mole fraction inequalities normalized by the daily UA variation (interquintile range)	eq S2
ΔXCO	XCO minus the daily UA-level twentieth percentile of XCO	–
ΔXNO_2_	XNO_2_ minus the daily UA-level twentieth percentile of XNO_2_	–
ΔXNO_2_/ΔXCO	TROPOMI NO_2_/CO enhancement ratios based on the slope of their reduced major-axis regression	–
ΔNO_2_/ΔCO	NO_2_/CO enhancement ratios (general notation)	–
NO_2_*	Measured surface in situ NO_2_ mixing ratios	–
NO_2_*/CO	Measured surface in situ NO_2_/CO ratios based on the slope of their reduced major-axis regression	–
E_NO2_/E_CO_	NEI20 NO_2_/CO emission ratios based on division	eq S5

Race and ethnicity data are from the 2016–2020 American Community Survey (ACS): 5-year data set.^57^ The ACS is affected by sampling and nonsampling errors, with errors caused by differential population group sampling rates, which are corrected through a nonpublic weighting algorithm that prioritizes accuracy over precision.^58,59^ The resulting imprecision can be large and is managed by census tract aggregation. The 2016–2020 5-year ACS data set used here is slightly misaligned temporally with the TROPOMI observations. We do not account for population changes by projecting the ACS data set into the future, as such attempts have been shown to yield large errors.^60^ Inequality trends over time have been shown to be almost entirely explained by air quality changes rather than migration.^8^

### 2.3 ΔXNO_2_/ΔXCO

TROPOMI ΔXNO_2_/ΔXCO are calculated daily as the slope of population-weighted census tract-scale ΔXCO and ΔXNO_2_ with a reduced major-axis regression in June–August.^13,61^ ΔXCO and ΔXNO_2_ are defined as XCO and XNO_2_ minus the same daily UA-level twentieth percentile values used to compute relative ΔXCO and ΔXNO_2_ inequalities. There is no standard approach to quantifying space-based enhancements to derive ΔXNO_2_/ΔXCO. Our approach means ΔXNO_2_/ΔXCO are more comparable to the reported XCO_norm_ and XNO_2,norm_. For example, Silva and Arellano^34^ assumed a Gaussian distribution of TROPOMI columns and identified enhancements as observations great than 2σ that distribution, Lama et al.^12^ used city-specific radii and computed enhancements based on concentrations across urban gradients, and MacDonald et al.^13^ identified enhancements using a fit based on specific XCO and XNO_2_ percentiles.

We focus on ΔXNO_2_/ΔXCO in June–August for two reasons. First, clear skies and snow-free scenes are most common in the summer, with fewer days with sufficient observational coverage in winter. Second, trace-gas enhancement ratios correspond to emission ratios when temporal variability in concentrations (C) and emissions (E) are correlated, ∂C/∂t ≈ E­(t). These conditions are typically best approximated in summertime when the atmosphere is less stable (mixing is efficient) and the NO_2_ chemical lifetime is short, such that the mass balance of primary species is driven by emissions and multiple primary trace species are correlated.^62^ Therefore, our ΔXNO_2_/ΔXCO analysis is biased toward summertime emissions patterns, which we discuss below. Lama et al.^12^ and MacDonald et al.^13^ also reported ΔXNO_2_/ΔXCO only for June–August. We exclude from the mean ΔXNO_2_/ΔXCO on days below TROPOMI coverage thresholds (Tables S3 and S4) and ΔXNO_2_/ΔXCO with *r* < 0 and/or that are statistically insignificant (*p* ≥ 0.050).^13^ This removes ∼60% of days on average across the UAs (Table S6). Because we are expressly interested in understanding the sources that cause inequalities, we focus on ΔXNO_2_/ΔXCO on days with significant absolute XNO_2_ or XCO inequalities. It is also informative to know ΔXNO_2_/ΔXCO on days without XNO_2_ and XCO inequalities, and we report ΔXNO_2_/ΔXCO on those days separately (Table 3). Uncertainties in ΔXNO_2_/ΔXCO are 1σ standard error of the mean, with σ derived from the mean daily standard error in slopes. This assumes the precision in the mean of daily ΔXNO_2_/ΔXCO scales inversely with the square root of the number of days in the average, accounting for differences in sampling statistics. ΔXNO_2_/ΔXCO are temporally variable (σ of 4–10%), as observed through other measurement approaches.^14^ Percent differences for Black, Latino, and Asian residents relative to non-Hispanic/Latino white residents have uncertainties of 1%, rounded up to the nearest integer and propagated from the 1σ standard error of the mean based on the square root of 70–322 days with quality-controlled ΔXNO_2_/ΔXCO observations (Table 3). For ΔXNO_2_/ΔXCO on weekdays and weekends separately, the numbers of days range 39–183 and 11–66, respectively (Table 3).

### 2.4 Surface Measurements

Hourly CO and NO_2_* mixing ratios are centralized in the U.S. EPA Air Quality System.^63^ Relevant measurement details are described in SI Appendix 3, including the known positive interference in NO_2_ chemiluminescence detection following catalytic decomposition of NO_2_ to NO that leads us to use the term NO_2_*.^64–67^ EPA-designated near-roadway monitors are excluded, as they are subject to hyperlocal effects. We calculate daily mean daytime (12–3 pm LT) CO and NO_2_* corresponding to the TROPOMI overpass time, which is also when the ABL tends to be well-developed, on days meeting TROPOMI coverage thresholds. We compare the slope of the correlation of CO and NO_2_* and TROPOMI ΔXNO_2_/ΔXCO. The locations with CO and NO_2_* measurements and their observation periods are given in Figure S4 and Table S7. We compare surface CO to TROPOMI XCO in the Los Angeles–Riverside U.S.-Census Core-Based Statistical Area (CBSA), a larger domain encompassing the Los Angeles–Riverside UA, that includes as many as 25 CO monitors. We compare NO_2_* and TROPOMI XNO_2_ in the two most-densely monitored CBSAs, Los Angeles–Riverside and Houston, which have 31 and 16 NO_2_ monitors, respectively (Table S8).

Hourly surface wind and temperature data were obtained from the Automated Surface Observing System (ASOS) and Automated Weather Observing System (AWOS).^68^ We calculate daily mean daytime (12–3 pm LT) wind speeds and air temperatures and daily median daytime wind directions using all available ASOS stations in each UA (Figure S5).

### 2.5 National Emissions Inventory (NEI)

The EPA produces annual CO and NO_*x*_ emissions estimates as part of its NEI; we use the 2020 NEI (NEI20).^23^ NEI on- and off-road vehicle CO and NO_*x*_ emissions are provided at the county level and individual stationary sources have latitude and longitude coordinates. The county scale is not sufficiently resolved spatially to distinguish aggregate census tract unit inequalities. Researchers and regulators may downscale the NEI for their modeling and decision-making or use higher-resolution inventories created by scientists outside the EPA; however, the county-level NEI remains an important policy-relevant tool as is, and a comparison to census tract-scale TROPOMI ΔXNO_2_/ΔXCO potentially informative for understanding NEI errors by users. To make this comparison, we average NEI20 on- and off-road vehicle CO and NO_*x*_ emissions to census tracts using area weighting and sum stationary sources within census tract boundaries. NO_*x*_ emissions are divided by a factor of 1.32 to convert to an effective NO_2_ emission rate,^13,69,70^ which situates our results in context of the body of literature. We discuss uncertainties associated with using a constant value for NO_*x*_/NO_2_ below. The NEI is not disaggregated by weekdays/weekends, seasonally, or by hour of day, which is a limitation. While we do not expect the spatial arrangement of sources to vary temporally, as the urban built environment is fixed, we do expect temporal variability in their magnitude. For example, freeways do not move with time, but traffic density and composition, engine demands, and fuels do. For stationary sources, Demetillo et al.^4^ found monthly resolved NEI point source NO_*x*_ emissions in July and January varied by ∼ 5% in the NEI (2017) in UAs across the U.S. Using the Fuel-based Inventory for Vehicle Emissions (FIVE18–19),^71–73^ Demetillo et al.^4^ reported a 60% reduction in NO_*x*_ emissions from on-road HDDVs weekdays to weekends, with similar emissions from on-road gasoline-powered vehicles. Uncertainties in time and space in our interpretation of differences between the NEI20 and ΔXNO_2_/ΔXCO are discussed below. To be consistent with ΔXCO and ΔXNO_2_, CO and NO_2_ emissions are reported on a molar basis, equal to their emission rates divided by their molar masses.

## 3 Results and Discussion

### 3.1 Census Tract-Scale CO and NO_2_ Inequalities

The atmospheric lifetime of CO is sufficiently long that CO spatiotemporal variability in the ABL is small relative to the full VCDs. While the sensitivity of TROPOMI is lowest near the surface,^12,74^ CO and NO_2_ are more evenly distributed in the free troposphere than the ABL on average. Census tract-scale heterogeneities in CO and NO_2_, which have atmospheric lifetimes longer than dilution time scales, are mostly a function of atmospheric dispersion rather than chemical loss.^24^ This causes steep spatial gradients horizontally and vertically that are not maintained above the ABL. Secondary production of CO affects intraurban variability, but on wider spatial scales than dispersion, with CO production occurring as fast as 1–3 h from reactive organic compounds.^14^ Intraurban CO variability is detected by TROPOMI (Figure 1 and Figure S1), and we report statistically significant absolute inequalities in XCO and CO VCDs and XNO_2_ and NO_2_ TVCDs for Black, Latino, and Asian population groups compared to the non-Hispanic/Latino white group in most UAs (Table S9). There is consistency between the most-exposed race-ethnicity group for CO and NO_2_, e.g., Black residents in New York City–Newark and Latino residents in Los Angeles–Riverside and Phoenix. Absolute XCO and XNO_2_ inequalities are strongly correlated across all UAs (*r* = 0.89) (Figure 2a), providing the first direct satellite evidence of unequal cumulative exposures in combustion air pollution mixtures.

**Figure 1:**
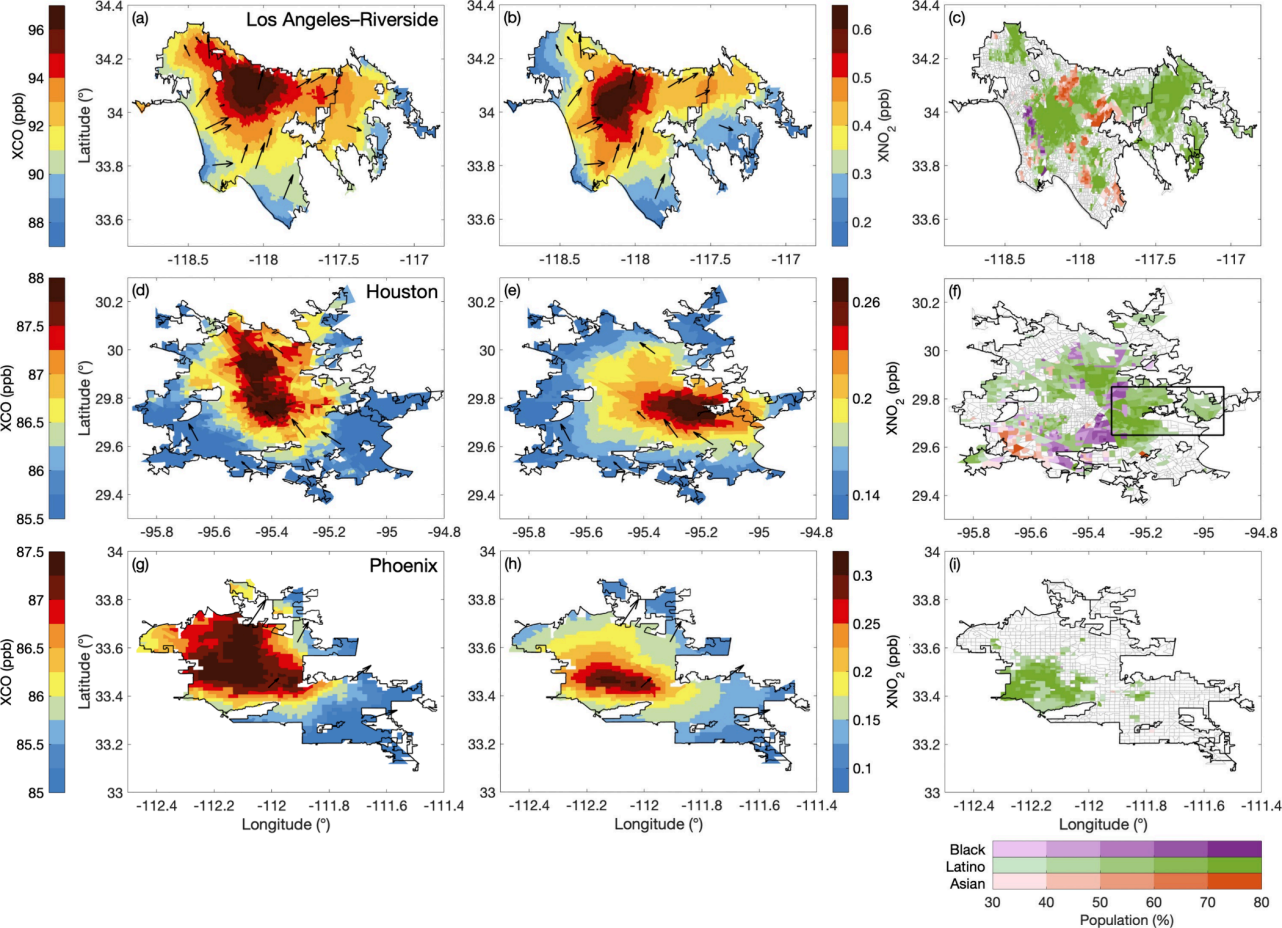
Mean daily census tract-scale XCO and XNO_2_ over June 2018–February 2023 (excluding March 2020–February 2021) in Los Angeles–Riverside (a–c), Houston (d–f), and Phoenix (g–i), with Black (purple), Latino (green), and Asian (orange) populations (%) in tracts where they are the largest race-ethnicity group. Mean daytime (12–3 pm LT) surface wind fields (black vectors) over the same period are shown. Thick black outlines are UA boundaries and the Houston Ship Channel (box). TROPOMI observations and demographics in Chicago, New York City–Newark, and Atlanta are available in Figure S1.

**Figure 2:**
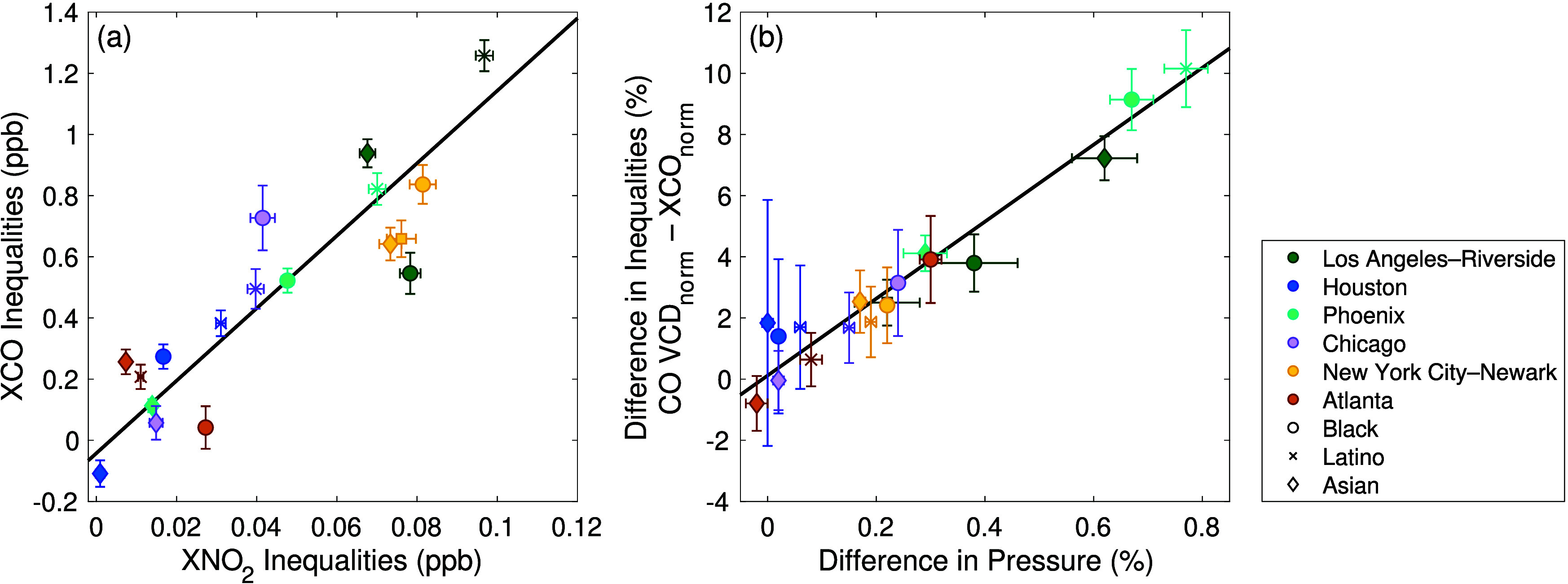
Annual mean daily census tract-scale absolute XCO and XNO_2_ inequalities (ppb) for Black (○), Latino (×), and Asian (◇) residents in Los Angeles–Riverside, Houston, Phoenix, Chicago, New York City–Newark, and Atlanta (a). The reduced major-axis regression line (black) is *y* = 11.9*x* – 0.04 with *r* = 0.89. Differences between inequalities in CO VCD_norm_ and XCO_norm_ (percentage points) and population-weighted surface pressures (b); *y* = 12.6*x* + 0.12 and *r* = 0.97. Error bars are uncertainties based on standard errors of the mean, and, for NO_2_, often not large enough to be seen behind the data markers.

A common approach to describing surface-level CO variability is to focus on XCO above an empirically determined tropospheric background.^13^ We report relative inequalities in XCO and CO VCDs equal to absolute inequalities normalized by the interquintile range in all daily census tract-scale observations, termed XCO_norm_ and CO VCD_norm_ (eq S2). In so doing, we focus on differences in the distribution of CO within UAs, as all urban population groups experience elevated CO pollution, without attempting to define a tropospheric CO background. This metric describes differences in exposures as a percentage of the urban variability. Mean daily inequalities in XCO_norm_ are statistically significant for some groups in some UAs, e.g., in Los Angeles, Phoenix, New York City, and Chicago, where they are 7–11% for Black, 7–15% for Latino, and 1–11% for Asian residents compared to non-Hispanic/Latino white residents (Table 2). However, in Houston and Atlanta, inequalities in XCO_norm_ are near or indistinguishable from zero. If we instead look at the distribution of the highest XCO within each UA, those XCO columns are more common in tracts with larger populations of Black, Latino, or Asian residents in some UAs. For example, inequalities in frequency of days with census tract-scale XCO above the median in Los Angeles, Phoenix, New York City, and Chicago are 7–17% for Black, 5–21% for Latino, and −2–16% for Asian residents, with much smaller differences between groups in Houston and Atlanta (−5–5%) (Table S10). If we were to report relative inequalities in XCO and CO VCDs as percent differences equal to absolute inequalities divided by the mean of their population-weighted CO values (eq S3), relative inequalities would be almost fully obscured (Table 2). This is because the daily XCO variation across each UA is typically small compared to the full atmospheric columns (XCO and CO VCDs) (Figure S3).

**Table 2:** Mean Daily Census Tract-Scale CO and NO_2_ Inequalities (%) over June 2018–February 2023 (excluding March 2020–February 2021) for Black, Latino, and Asian Population Groups Relative to the Non-Hispanic/Latino White Population Group in Each UA^a^

	**Inequalities (%)**							
	**Relative to the urban variability**		**Relative to the full atmospheric column**		**Relative to the urban variability**		**Relative to the tropospheric column**	
**Population Group**	**CO dry-air mole fraction (XCO**_**norm**_ ^b^ **)**	**CO column density (CO VCD**_**norm**_ ^b^ **)**	**CO dry-air mole fraction (XCO** ^c^ **)**	**CO column density (CO VCDs** ^c^ **)**	**NO**_**2**_ **dry-air mole fraction (XNO**_**2,norm**_ ^b^ **)**	**NO**_**2**_ **column density (NO**_**2**_ **TVCD**_**norm**_ ^b^ **)**	**NO**_**2**_ **dry-air mole fraction (XNO**_**2**_ ^c^ **)**	**NO**_**2**_ **column density (NO**_**2**_ **TVCDs** ^c^ **)**
**Los Angeles–Riverside**								
Black	7	11	0.6	1	27	27	20	21
Latino	15	17	1.4	1.5	34	35	27	27
Asian	11	18	1	1.7	23	23	18	19
**Houston**								
Black	3 ± 2	4	0.3	0.3	14	14	9	9
Latino	5 ± 2	7	0.4	0.5	27	27	17	17
Asian	–5 ± 4	–3 ± 2	–0.2	–0.2	–1	–1	–2	–2
**Phoenix**								
Black	10	19	0.6	1.1	35	36	22	23
Latino	15	25	0.9	1.5	52	53	31	31
Asian	2	6	0.1	0.4	10	10	7	7
**Chicago**								
Black	10	13	0.7	0.9	23	23	16	16
Latino	7	9	0.5	0.6	21	21	15	15
Asian	1	1	0	0	8	8	4	4
**New York City–Newark**								
Black	11	13	0.8	1	28	28	24	24
Latino	8	10	0.7	0.8	26	26	21	21
Asian	8	11	0.6	0.8	26	26	22	22
**Atlanta**								
Black	1	5	0	0.3	33	33	19	19
Latino	3	4	0.2	0.3	13	13	8	9
Asian	4	3	0.3	0.2	9	9	6	6

a Results compared based on dry-air mole fractions versus column densities and urban-variability versus column normalized observations. Uncertainties are the 1σ standard error of the mean. Uncertainties are typically 1% for XCO_norm_, CO VCD_norm_, XNO_2,norm_, NO_2_ TVCD_norm_, XNO_2_, and NO_2_ TVCDs and 0.1% for XCO and CO VCDs and are only displayed if different from these values.

b Relative inequality is defined as absolute inequality divided by the UA interquintile range, multiplied by 100 (eq S2).

c Relative inequality is defined using percent difference, which is equal to the absolute inequality divided by the mean of the two values being compared, multiplied by 100 (eq S3).

Differences in inequalities in XCO_norm_ and CO VCD_norm_ (Table 2) are explained by intraurban differences in mean population-weighted residential elevation (Figure 2b). Black and Latino residents are known to experience more frequent flooding as they often live at lower elevations within cities.^75,76^ All else being equal, taller VCDs contain more air, including more CO. We compare inequalities in XCO_norm_ and CO VCD_norm_ over a range of intraurban elevation differences using population-weighted surface pressures calculated from the hydrostatic equation (eq S4) from the NASA Shuttle Radar Topographic Mission 90-m Digital Elevation Database.^77^ The differences in inequalities and population-weighted surface pressures are strongly correlated (*r* = 0.97). We conclude that inequalities based on CO VCDs confound population-weighted differences in elevation, with XCO a more accurate metric for representing near-surface CO spatial heterogeneities, especially where elevation differences within cities are large.

Inequalities in XNO_2,norm_ and NO_2_ TVCD_norm_ are also larger than inequalities in XNO_2_ and NO_2_ TVCDs (Table 2). In cities, a major portion of NO_2_ variability in the ABL on neighborhood scales is caused by NO_2_ dispersion away from NO_*x*_ sources.^5,24,25^ While NO_*x*_ emissions are predominantly nitric oxide (NO),^78^ NO and NO_2_ reach steady state in ∼100 s (faster than time scales of dispersion), with NO/NO_2_ determined by local ozone (O_3_) concentrations and NO_2_ photolysis rates. Therefore, daytime NO_2_ spatial heterogeneities in the very near field of sources are driven by dilution into the urban background of NO_2_ and O_3_ titration by high NO. While NO_2_ is not a primary pollutant per se, it behaves as a pseudoprimary pollutant, varying exponentially on length scales of hundreds of meters to 1–2 km away from NO_*x*_ sources,^24,25^ and can be treated as a proxy for NO_*x*_ in urban areas. NO_2_ is also present away from sources in the free troposphere and rural ABL. While NO_2_ mixing ratios aloft may be just a few ppt, TVCDs are vertically integrated, and low NO_2_ concentrations in the free troposphere contribute to measured TVCDs over polluted cities.^79–88^ Relative inequalities in NO_2_ TVCD_norm_ are on average seven percentage points higher than those derived from NO_2_ TVCDs. Unlike CO, relative inequalities in XNO_2,norm_ and NO_2_ TVCD_norm_ are equal to within associated uncertainties (propagated from 1σ standard errors of the mean). We attribute this to the shorter lifetime of NO_2_, with time scales of NO_2_ chemical loss and convective mixing being competitive,^89^ which causes a larger portion of NO_2_ columns to be lower in the ABL compared to CO. As a result, differences in atmospheric column height have less influence over census tract-scale NO_2_ inequalities. Inequalities in XNO_2,norm_ for Black, Latino, and Asian residents are 14–35%, 13–52%, and – 1–26%, respectively (Table 2).

Census-tract scale inequalities in TROPOMI NO_2_ TVCDs have been compared against spatially comprehensive airborne remote sensing that resolves length scales of dispersion;^5,6,51^ however, these observations are not available for CO. Routine CO monitoring is sparser than for NO_2_, with fewer than 300 CO regulatory monitors across the U.S. and only a handful in cities outside of California. Additionally, regulatory CO instruments often report a coarse CO signal response. This limits analysis of distance-dependent column-surface correlations previously used to demonstrate spatial similarities in NO_2_ TVCDs and NO_2_*.^4,5,40^ We discuss TROPOMI CO and NO_2_ inequalities in the context of their observed and known atmospheric variability and documented uncertainties in inequalities in NO_2_ TVCDs. In this way, we evaluate census tract-scale TROPOMI CO inequalities and explore controls over their severity, describing XCO and XNO_2_ inequalities on weekdays and weekends, with variations in surface winds and air temperatures, and seasonally.

Weekday-weekend air quality differences in U.S. cities are largely driven by weekend reductions in NO_*x*_ emissions from HDDVs that transport commercial goods. Emissions from industrial point sources, power plants, and passenger vehicles, which are mostly gasoline-powered in the U.S., exhibit much less weekday-weekend variability.^67,90^ While the timing of vehicle emissions may shift, the effect on composition is minor compared to the change in mass.^91^ Off-road diesel NO_*x*_ emissions vary on weekdays and weekends but are a comparatively smaller contribution to urban NO_2_.^4^ While HDDV exhaust aftertreatment devices for NO_*x*_ are becoming more prevalent,^88^ HDDVs remain a major portion of NO_2_ in U.S. cities and census tract-scale NO_2_ inequalities.^4^ Additionally, NO_2_ is a major chemical control over hydroxyl radical (OH) concentrations that affect CO production and CO and NO_2_ lifetimes. CO and NO_2_ concentrations can also vary because of this perturbation to OH, although the effect is smaller and the dependence of OH on NO_2_ is nonlinear.^92,93^ We compare CO and NO_2_ inequalities on weekdays (Tuesday–Friday) and weekends (Saturday–Sunday) (Figure 3). Annual absolute XNO_2_ inequalities are 36 ± 8% lower on weekends than weekdays on average, based on the slope of reduced major-axis regression across all UAs and population groups (Figure 3). This is comparable with Demetillo et al.,^4^ who reported inequalities in oversampled TROPOMI NO_2_ TVCDs based on race-ethnicity and median household income decreased on weekends by 37% (summer) and 32% (winter) across 52 U.S. cities from a 60% reduction in HDDV emissions. Correspondingly, absolute XCO inequalities are not statistically significantly different on weekdays and weekends (Figure 3). This is evidence both that the sources contributing to CO inequalities are similar on weekdays and weekends and that TROPOMI detects physically plausible CO variability. While the lifetime of CO is ∼1 month, the ABL residence time is on the order of 1 day, and weekday-weekend CO variability reflects emissions patterns^94^ consistent with observations from roadside and tunnel studies of fuel-based emissions measurements.^95^

**Figure 3:**
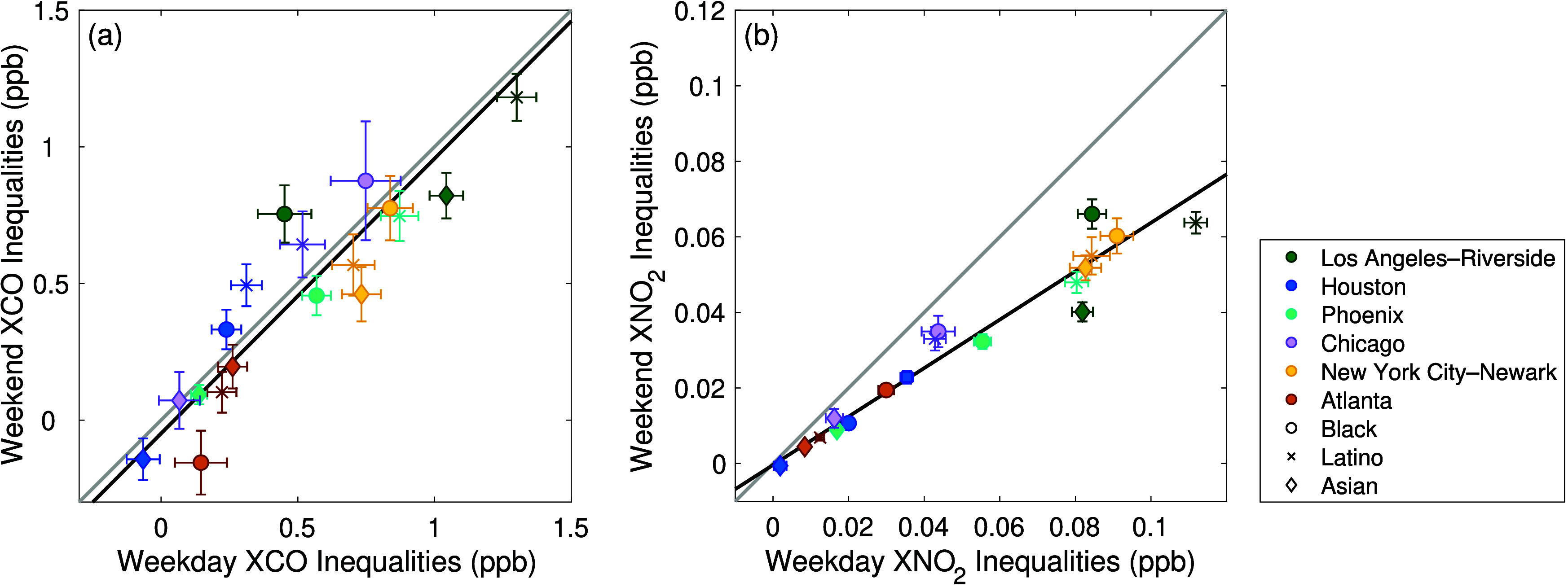
Absolute XCO (a) and XNO_2_ (b) inequalities (ppb) on weekdays and weekends for Black (○), Latino (×), and Asian (◇) residents in Los Angeles–Riverside, Houston, Phoenix, Chicago, New York City–Newark, and Atlanta. Pearson correlation coefficients are 0.91 (XCO) and 0.97 (XNO_2_). The black line is the reduced major-axis regression: *y* = 1.01*x* – 0.05 (XCO) and *y* = 0.64*x* – 0.00 (XNO_2_). The gray line is 1:1. Error bars are uncertainties based on standard errors of the mean.

CO and NO_2_ inequalities vary with both emissions source strength and atmospheric dispersion as CO and NO_*x*_ emissions are diluted into the urban background.^24,25^ While TROPOMI pixels are larger than dispersion length scales, emissions sources are often clustered into source regions where their gradients aggregate.^4,6,51,96^ As a result, absolute XCO inequalities are expected to vary inversely with surface wind speeds, as calm, stable atmospheric conditions reduce mixing and concentrate pollutants near sources.^6^ Both absolute and relative CO and NO_2_ inequalities are higher on days with slow (below median) than fast (above median) daytime winds for most metrics (Table S11). Absolute XCO and XNO_2_ inequalities are also significantly (*p* < 0.050) negatively correlated with daytime (12–3 pm LT) UA-level surface wind speeds for almost all metrics based on their Spearman’s rank correlation coefficient, a nonparametric version of the Pearson correlation coefficient (Table S12). That said, associations between absolute XNO_2_ inequalities and surface wind speeds are consistently stronger than for XCO inequalities, which, although significant, are generally weak. This suggests intraurban spatial distributions of CO are affected by an additional term, which we hypothesize is secondary CO production. Evidence for this includes absolute XCO inequalities that are generally higher on days with cooler (below median) temperatures annually (Table S13), i.e., conditions associated with less photochemical oxidation and lower emissions of reactive CO precursors,^14,26,97^ and inequalities in XCO_norm_ that are mostly larger in winter (December–February), 7–19% for Black, 5–28% for Latino, and 1–12% for Asian compared to non-Hispanic/Latino white residents, than summer (June–August) (Table S14). Seasonal differences in ABL height do not affect results based on columns, at least not directly. XCO and XNO_2_ inequalities are also more strongly correlated in winter (*r* = 0.92) than summer (*r* = 0.79) (Figure S6). Relatedly, we observe that XCO is distributed spatially more broadly than XNO_2_ and elevated in the direction downwind of the NO_2_ plume (Figure 1). NO_2_ concentrations are limited by the short lifetime of NO_2_, while CO is produced along the plume trajectory. This is apparent in Houston, where XCO is elevated downwind, but not over, the Houston Ship Channel, an industrial center with high emissions of reactive organic compounds.^40,98^

### 3.2 NO_2_/CO Enhancement Ratios

Trace gas enhancement ratios give insight into the causes of urban air pollution inequalities because different source types emit pollutants in characteristic ratios.^9^ NO_2_/CO emitted from different combustion sources varies substantially (Table S15), with the NEI predicting gasoline vehicles and nonroad mobile equipment having the lowest NO_2_/CO except for fires.^14,99^ NO_2_/CO enhancement ratios are typically computed as the regression slope of paired CO and NO_2_ (or NO_*x*_ or NO_*y*_ ≡ NO_*x*_ + its higher oxides) minus their observationally determined background values. The interpretation of NO_2_/CO is complicated as atmospheric conditions and chemistry are dynamic and NO_2_/CO highly variable in space and time.^13–15,38^ This especially affects analyses that use measurements collected far away from tailpipes and exhaust stacks, e.g., satellite and aircraft observations. Still, TROPOMI CO and NO_2_ have been used to infer source-specific NO_2_/CO in urban and biomass-burning plumes and generate top-down constraints on inventories.^12,13,34,100^ While plume dilution does not directly affect NO_2_/CO, conditions that mix or stagnate plumes can obscure NO_2_/CO, such that observed changes in concentrations are not proximate for changes in emissions. In particular in winter, longer NO_2_ lifetimes cause NO_2_ to dislocate from emitters, integrating NO_*x*_ sources spatiotemporally,^101^ and stable wintertime atmospheric conditions reduce dispersion and vertical mixing, leading to wider areas of influence near sources.^102^ As a result, we focus on TROPOMI ΔXNO_2_/ΔXCO in June–August, when satellite observations are less affected by clouds/snow, and consistent with the work of others based on TROPOMI.^12,13^ Our analysis misses seasonal variability in ΔXNO_2_/ΔXCO, including sector-level seasonal trends in emissions from power generation^103^ and vehicles^38^ (although fuel sales are similar seasonally^72^).

ΔXNO_2_/ΔXCO are generally higher in Black, Latino, and Asian neighborhoods than primarily non-Hispanic/Latino white census tracts (Table 3). This is direct observational evidence that these groups are disproportionately exposed to higher CO and NO_2_ (Table 2) and systematically exposed to different mixtures of pollution characterized by sources with higher NO_2_/CO. Absolute and relative differences in population-weighted ΔXNO_2_/ΔXCO on the subset of days with CO and/or NO_2_ inequalities are comparable to all days because days with inequalities are so frequent. Population-weighted differences in ΔXNO_2_/ΔXCO on days without CO and/or NO_2_ inequalities with race-ethnicity are close to zero, although statistics are limited (Table 3). Higher aggregate NEI emissions-based NO_2_/CO (E_NO2_/E_CO_) (eq S5) are caused by HDDVs (Table S15). Population-weighted differences in ΔXNO_2_/ΔXCO reinforce conclusions from Demetillo et al.^4^ that HDDVs drive urban NO_2_ disparities. Using ΔXNO_2_/ΔXCO, we also show that Black, Latino, and Asian residents are not just exposed to higher NO_2_ levels, they are disproportionately exposed to HDDV exhaust such that even at the same NO_2_ levels, the air mixture is likely more dangerous because of HDDVs’ toxic coemissions. In the context of CO and NO_*x*_ emissions trends, because CO emissions are decreasing at a faster rate than NO_*x*_,^28^ non-Hispanic/Latino white residents have benefited more on average from those controls than groups more affected by high NO_2_/CO sources.

**Table 3:** Mean Daily Census Tract-Scale ΔXNO_2_/ΔXCO (ppb ppb^–1^) on Weekdays (Tuesday–Friday) and Weekends (Saturday–Sunday) in Summer (June–August) with the Number of Days in the Estimate (Table S6), Corresponding Percent Changes; Percent Differences in ΔXNO_2_/ΔXCO Relative to the Non-Hispanic/Latino White Group on Weekdays, Weekends, and Days with and without Absolute XCO and/or XNO_2_ Inequalities, with the Number of Days Meeting that Condition (*N*); Weekday TROPOMI ΔXNO_2_/ΔXCO and Percent Differences Relative to the Non-Hispanic/Latino White Group with the CO and NO_2_ Averaging Kernels Applied; and NEI20 E_NO2_/E_CO_ and Percent Differences Similarly^a^

**TROPOMI ΔXNO**_**2**_ **/ΔXCO (ppb ppb** ^**–1**^ **)**				**TROPOMI ΔXNO**_**2**_ **/ΔXCO Percent Differences Relative to Non-Hispanic/Latino Whites (%)**				**TROPOMI ΔXNO**_**2**_ **/ΔXCO with CO and NO**_**2**_ **averaging kernels**		**NEI20 E**_**NO2**_ **/E**_**CO**_	
**Population Group**	**Weekday**	**Weekend**	**Weekday– Weekend (%)** ^b^	**Weekday** ^b^	**Weekend** ^b^	**Days with Inequalities (** *N* **)** ^b^	**Days without Inequalities (** *N* **)** ^b^	**Weekday**	**Percent Difference (%)** ^b^	**mol mol^–1^**	**Percent Difference (%)** ^b^
**Los Angeles–Riverside**	(*N* = 183)	(*N* = 66)									
Black	0.0274	0.0164	–50	12 ± 1	16 ± 1	15 ± 1 (275)	–9 ± 1 (14)	0.0247	6 ± 1	0.0837	15 ± 3
Latino	0.0320	0.0195	–48	27 ± 1	33 ± 1	30 ± 1 (322)	– (0)	0.0291	23 ± 1	0.0839	15 ± 2
Asian	0.0315	0.0187	–51	26 ± 1	31 ± 1	29 ± 1 (318)	15 ± 1 (11)	0.0321	34 ± 1	0.0669	–8 ± 2
Non-Hispanic/Latino White	0.0242	0.0135	–56	–	–	–	–	0.0228	–	0.0723	–
**Houston**	(*N* = 63)	(*N* = 18)									
Black	0.0156	0.0114	–31	9 ± 1	6 ± 1	11 ± 1 (124)	–1 ± 1 (6)	0.0039	15 ± 1	0.0572	–22 ± 4
Latino	0.0173	0.0126	–31	16 ± 1	13 ± 1	15 ± 1 (120)	– (0)	0.0041	17 ± 1	0.0614	–15 ± 5
Asian	0.0140	0.0102	–32	–2 ± 1	–7 ± 3	2 ± 1 (70)	–7 ± 1 (58)	0.0035	5 ± 1	0.0581	–21 ± 4
Non-Hispanic/Latino White	0.0144	0.0106	–30	–	–	–	–	0.0033	–	0.0716	–
**Phoenix**	(*N* = 142)	(*N* = 55)									
Black	0.0242	0.0164	–38	36 ± 1	30 ± 1	35 ± 1 (249)	3 ± 2 (9)	0.0236	33 ± 1	0.0507	4 ± 11
Latino	0.0255	0.0167	–42	40 ± 1	31 ± 1	38 ± 1 (239)	–5 ± 11 (5)	0.0246	36 ± 1	0.0510	5 ± 10
Asian	0.0208	0.0140	–39	20 ± 1	15 ± 1	20 ± 1 (239)	4 ± 2 (35)	0.0206	18 ± 1	0.0492	1 ± 7
Non-Hispanic/Latino White	0.0171	0.0120	–35	–	–	–	–	0.0172	–	0.0487	–
**Chicago**	(*N* = 60)	(*N* = 31)									
Black	0.0185	0.0107	–54	–15 ± 1	2 ± 1	–8 ± 1 (110)	–24 ± 2 (18)	0.0041	0 ± 1	0.0622	12 ± 5
Latino	0.0230	0.0118	–64	6 ± 1	8 ± 1	7 ± 1 (148)	–4 ± 15 (3)	0.0045	5 ± 1	0.0698	24 ± 6
Asian	0.0209	0.0103	–68	–1 ± 1	–2 ± 2	1 ± 1 (93)	–5 ± 1 (52)	0.0043	6 ± 1	0.0545	–1 ± 4
Non-Hispanic/Latino White	0.0205	0.0104	–65	–	–	–	–	0.0041	–	0.0551	–
**New York City–Newark**	(*N* = 81)	(*N* = 31)									
Black	0.0337	0.0213	–45	14 ± 1	19 ± 1	16 ± 1 (142)	–11 ± 8 (1)	0.0087	19 ± 1	0.0532	7 ± 5
Latino	0.0336	0.0213	–45	14 ± 1	18 ± 1	16 ± 1 (138)	4 ± 3 (2)	0.0082	16 ± 1	0.0551	10 ± 4
Asian	0.0324	0.0210	–42	13 ± 1	19 ± 1	14 ± 1 (149)	– (0)	0.0082	20 ± 1	0.0520	4 ± 4
Non-Hispanic/Latino White	0.0284	0.0176	–47	–	–	–	–	0.0066	–	0.0498	–
**Atlanta**	(*N* = 39)	(*N* = 11)									
Black	0.0104	0.0088	–17	22 ± 1	28 ± 2	19 ± 1 (74)	5 ± 14 (3)	0.0036	23 ± 1	0.0543	12 ± 10
Latino	0.0094	0.0060	–44	14 ± 1	9 ± 3	12 ± 1 (79)	1 ± 1 (5)	0.0030	14 ± 1	0.0486	1 ± 14
Asian	0.0088	0.0064	–31	7 ± 1	15 ± 3	8 ± 1 (76)	7 ± 3 (12)	0.0027	8 ± 1	0.0491	2 ± 14
Non-Hispanic/Latino White	0.0081	0.0055	–38	–	–	–	–	0.0023	–	0.0481	–

a Uncertainties are propagated as derived from 1σ/*N* ^1/2^.

b Percent difference equal to the difference between two values divided by the mean of the two values being compared, multiplied by 100.

Population-weighted ΔXNO_2_/ΔXCO decrease substantially from weekdays to weekends for all population groups because of reduced HDDV traffic (Table 3). In some UAs, weekend declines in ΔXNO_2_/ΔXCO are similar between race-ethnicity groups, e.g., Houston. In other UAs, HDDVs are more persistent on weekends, with smaller decreases in Black, Latino, and Asian neighborhoods than for non-Hispanic/Latino white residents. Absolute differences in ΔXNO_2_/ΔXCO for Black, Latino, and Asian population groups compared to non-Hispanic/Latino white residents are 36% lower on weekends than weekdays based on the slope of reduced major-axis regression across all UAs (*r* = 0.94) (Figure S7); at the same time, relative differences are similar (Table 3). Even though the magnitude of HDDV emissions is reduced on weekends, relative inequalities remain because disparities in source mixtures are maintained. This means HDDVs are a larger source of combustion pollution in primarily Black, Latino, and Asian census tracts on both weekdays and weekends.

Uncertainties in TROPOMI ΔXNO_2_/ΔXCO have previously been characterized on city scales. Errors are affected by background selection methodology and CO and NO_2_ chemistry, and comparisons between ΔXNO_2_/ΔXCO and inventories (as E_NO2_/E_CO_) require an estimate of NO_*x*_/NO_2_. TROPOMI is also differentially sensitive to CO and NO_2_ vertically. The resulting errors combine to bias UA-level TROPOMI ΔXNO_2_/ΔXCO low.^12,13^ Our discussion here focuses on how errors influence estimates of intraurban differences in ΔXNO_2_/ΔXCO, not absolute city-wide ΔXNO_2_/ΔXCO. First, while we defined ΔXNO_2_/ΔXCO using enhancements above the daily twentieth percentiles used to compute the interquintile range for XCO_norm_ and XNO_2,norm_ inequalities, we also tested (a) seasonal and annual average domain-wide backgrounds as either the fiftieth or seventy-fifth XCO and XNO_2_ percentiles as done in MacDonald et al.^13^ or (b) UA-specific percentiles constrained to produce equivalent absolute inequalities in both XCO and ΔXCO and XNO_2_ and ΔXNO_2_.^53^ The background selection approach minimally affected ΔXNO_2_/ΔXCO and yielded consistent relationships between population groups. While ΔXNO_2_/ΔXCO are not impacted by CO or NO_2_ backgrounds when vertically uniform, the regression does not account for background values that vary independently. Simon et al.^14^ tested the sensitivity of ΔNO_2_/ΔCO to in situ aircraft sampling within ABL and free troposphere over Baltimore, Maryland, finding that when measurements from higher altitudes were included in the regression, ΔNO_2_/ΔCO systematically decreased. Simon et al.^14^ emphasized the importance of restricting regressions to data collected within the ABL, as measurements aloft incorporate air from the free troposphere where variability in background CO can be large relative to NO_2_. This is not possible with columns but would affect population groups largely evenly within UAs.

Second, OH chemistry converts NO_*x*_ to higher nitrogen oxides unmeasured by TROPOMI that deposit more readily than NO_2_. In-situ analyses often use NO_*y*_ instead of NO_2_ or NO_*x*_, and studies with TROPOMI have estimated NO_2_ chemical lifetimes to correct for NO_2_ loss across the urban domain. The short NO_2_ lifetime is an asset when aggregating across census tracts, with no correction required to interpret intraurban differences. OH also eventually produces CO in the oxidation of most organic compounds, lowering ΔXNO_2_/ΔXCO as relevant to E_NO2_/E_CO_. Simon et al.^14^ concluded that CO production influenced the observed ΔNO_2_/ΔCO when emissions of reactive biogenic organic compounds were high even at low altitudes within the ABL over a major freeway. This secondary CO potentially contributes to inequalities in XCO_norm_, e.g., in Houston, but should affect urban residents more, although not fully, evenly spatially.

Third, we are inferring differences in emissions ratios, but NO_2_ is emitted as NO_*x*_. While O_3_ is a major control on NO_*x*_/NO_2_, O_3_ is secondary, intermediately long-lived, and not associated with neighborhood-level inequalities.^8,104^ TROPOMI misses the portion of NO_*x*_ stored as NO as the system reaches steady state. This is not accounted for by using a constant NO_*x*_/NO_2_ and biases TROPOMI NO_*x*_ emissions estimates low.^22,105^ While beyond the scope of our analysis, others have shown that using a spatiotemporally varying coefficient function rather than a constant value reduces the associated bias in NO_*x*_ emissions estimates in the near sources.^105^ Demetillo et al.^5^ showed that NO_2_ as a proxy for NO_*x*_ is underestimated in small-area census units near NO_*x*_ sources. Because Black, Latino, and Asian residents are more likely to live both near NO_*x*_ sources and in smaller census tracts,^4–6,40^ intraurban population-weighted differences in ΔXNO_2_/ΔXCO are likely a lower bound.

Because CO is retrieved in the short-wave infrared, TROPOMI is more sensitive to CO than NO_2_ near the surface.^12^ TROPOMI CO and NO_2_ sensitivities can vary horizontally within cities. To test this, we divide daily census tract-scale ΔXCO and ΔXNO_2_ by their respective mean near-surface averaging kernels and recompute population-weighted ΔXNO_2_/ΔXCO (Table 3). We treat the near-surface CO averaging kernels as the mean of the first two levels, 500 and 1500 m above ground level (AGL), which are the available layers most likely within the summertime ABL, to reduce observed noise in the 500-m layer (Table S16; described below). We use the mean of the first five layers in the NO_2_ averaging kernels, corresponding to the atmospheric heights represented in the first two CO levels. In the lowest layer of the NO_2_ averaging kernel, TROPOMI is on average 2.9 ± 2.7% (±1σ standard deviation) and up to 9% more sensitive to NO_2_ in primarily Black, Latino, and Asian than non-Hispanic/Latino white census tracts, but differences average out across the first five layers (Table S16). While TROPOMI ΔXNO_2_/ΔXCO are similar or decrease when the averaging kernels are applied, relationships between groups are comparable (Table 3), indicating that differential TROPOMI CO and NO_2_ near-surface sensitivities do not drive population-weighted ΔXNO_2_/ΔXCO differences.

Lastly, we compare XCO and CO and/or XNO_2_ and NO_2_* as a function of observation separation distance and evaluate NO_2_/CO column-surface relationships. Los Angeles–Riverside is the only city in the analysis with enough CO monitors to compare XCO and CO, which we do across the full CBSA (Table S17). We repeat the analysis where NO_2_* monitors are sufficiently dense, which is in Los Angeles–Riverside and the Houston CBSA. Pearson correlation of XCO and CO mixing ratios exhibit *r* = 0.52 within 1 km of a monitor (based on tract center points), with *r* values decreasing by a factor of 2 at distances of 2–10 km, consistent with length scales of dispersion. XNO_2_ and NO_2_* are also best correlated within 1 km of a monitor (*r* = 0.66 in Los Angeles–Riverside and *r* = 0.74 in Houston), declining by 10–25% at 2–10 km. We use this comparison to test whether dividing by the near-surface averaging kernels adds noise to ΔXNO_2_/ΔXCO, as their application should not worsen column-surface agreement (Table S17). For CO, dividing by just the bottom layer worsens XCO and CO agreement, especially within 1 km of a monitor (*r* = 0.35). By using the mean of the first two layers, we derive similar correlations for co-located XCO and CO (*r* = 0.55), likewise strengthening gradients in *r* with distance. For NO_2_, results are similar when dividing by either the bottom or lowest five layers. To compare ΔXNO_2_/ΔXCO and NO_2_*/CO, we compute regression slopes of mean daytime (12–3 pm LT) surface NO_2_*/CO using reduced major-axis regression in June–August on days with TROPOMI observations in >20% of tracts within 5 km of that monitor and with significant slopes (*p* < 0.050) and *r* > 0 (Table S18). ΔXNO_2_/ΔXCO and NO_2_*/CO are moderately correlated (*r* = 0.41) across UAs, with requirements for co-located CO and NO_2_* measurements on days with ΔXNO_2_/ΔXCO reducing the data set size, especially at within 1 km of a monitor. While TROPOMI ΔXNO_2_/ΔXCO are generally lower than NO_2_*/CO, they agree to within an order of magnitude.

The NEI is a tool for decision-makers in the design and evaluation of air pollution mitigation strategies for regulatory compliance and related goals used in combination with a chemical transport model, with state and district-level agencies responsible for contributing emissions data to the NEI that is accurate and complete. Satellite ΔXNO_2_/ΔXCO have been used to validate inventories at city scales, showing E_NO2_/E_CO_ are higher than TROPOMI ΔXNO_2_/ΔXCO in cities globally, attributed to underestimates in bottom-up CO emissions, specifically in the Emissions Database for Global Atmospheric Research (EDGAR).^12,13^ A similar result has been reported using roof-top in situ observations in New York City: EDGAR CO emissions are biased low.^97^ City-average TROPOMI ΔXNO_2_/ΔXCO have not yet been compared to any version of the NEI; however, the prevailing understanding is that NEI CO emissions are overestimated by as much as a factor of 2 compared to observations,^38,71,101,106,107^ although others have reported smaller differences.^15,26,108,109^ We find TROPOMI ΔXNO_2_/ΔXCO are consistently lower than annual NEI20 E_NO2_/E_CO_, especially after dividing ΔXNO_2_ and ΔXCO by their near-surface averaging kernels (Table 3). The NEI is also known to overestimate mobile NO_*x*_ emissions,^81,110,111^ and we have not corrected ΔXNO_2_/ΔXCO for the portion of NO_2_ chemically converted to NO_*y*_ within city boundaries;^13^ therefore, we do not draw conclusions about absolute NEI errors city-wide. Instead, we focus on intraurban differences in population-weighted ΔXNO_2_/ΔXCO and NEI20 E_NO2_/E_CO_, which can reveal patterns in sources relevant to inventory accuracy. The comparison is influenced both by errors in the NEI20 and its lack of census tract-scale spatial resolution, which we use without downscaling, as the NEI20 is, as is, an official decision-making tool produced by and available to regulators from the EPA. We find the NEI20 underestimates the extent to which emissions are characterized by higher NO_2_/CO in Black, Latino, and Asian neighborhoods. Discrepancies are largest in Houston but generally observed across the major UAs except for in Chicago. There are distributive CO and NO_2_ inequalities with race-ethnicity in Chicago, and HDDVs are a known environmental justice issue locally. It could be that NO_2_ inequalities are driven by source density rather than composition, meaning low NO_2_/CO sources also overburden Chicago’s Black and Latino residents. However, because ΔXNO_2_/ΔXCO are biased low, it may also be that there is meaningful spatial variability in ΔXNO_2_/ΔXCO that is undetectable by TROPOMI. This article does not answer the question of whether the agreement between population-weighted ΔXNO_2_/ΔXCO and NEI20 E_NO2_/E_CO_ improves when the NEI20 is downscaled to a finer grid prior to census tract averaging. That is because downscaling is nontrivial and based on researchers’ or regulators’ decisions without EPA guidance. However, ΔXNO_2_/ΔXCO provide observational constraints on such future work and evidence that downscaling is necessary for modeling and decision-making related to environmental justice.

### 3.3 Implications

NO_2_ is a common surrogate for combustion mixtures in health research,^112^ and TROPOMI NO_2_ inequalities have been described in this context.^4,5^ We report satellite evidence of both CO inequalities and cumulative air pollution burdens, as the most exposed race-ethnicity groups for CO and NO_2_ are often the same. Because CO is long-lived in the atmosphere, census tract-scale TROPOMI CO inequalities require new approaches than those developed using NO_2_ TVCDs, with TROPOMI CO disparities influenced by other environmental inequalities, e.g., intraurban differences in residential elevation. Differences in population-weighted ΔXNO_2_/ΔXCO indicate that NO_2_ as a proxy for combustion pollution is differently true,^62^ with NO_2_ concentrations in primarily Black, Latino, and Asian neighborhoods representing combustion mixtures disproportionately affected by HDDVs, even on weekends. While incremental sector-based controls, especially on gasoline-powered vehicles with low NO_2_/CO, have not changed the underlying spatial distributions of sources and, therefore, exposure disparites,^8^ regulations on HDDVs, which have high NO_2_/CO, specifically would have environmental justice benefits.^5^ To eliminate disparities in combustion pollution exposures, politicians and other decision-makers must ultimately address disparities in source composition and not just emissions magnitude. However, inaccuracies in the NEI20 and/or errors caused by its county-level resolution could have the effect of directing attention and resources away from controls on HDDVs, even for regulators committed to equity. Where TROPOMI ΔXNO_2_/ΔXCO are constraints on the spatial distribution of emissions in inventories, as well as empirical models of neighborhood-level air quality for health research, they demonstrate the value of satellite instruments that measure multiple, related pollutants at neighborhood scales. Finally, TROPOMI ΔXNO_2_/ΔXCO observations are positioned to monitor the impacts of recent fleet electrification investments and new HDDV emissions controls including an 80% NO_*x*_ emissions reduction beginning in the 2027 model year^113^ on air pollution inequalities.

## Supplementary Material



## Notes

XCO and XNO_2_ in Chicago, New York City–Newark, and Atlanta; sensitivities of inequalities to pixels over water in New York City–Newark, pressure product, and to TROPOMI coverage and pixel size; summary of past research evaluating inequalities in TROPOMI NO_2_ TVCDs; workflow schematic; equations for population weighting, relative inequalities, the hydrostatic equation, and NEI emission ratios on a molar basis; XCO timeseries and the frequency of XCO observations below the twentieth and above the eightieth percentiles in Pheonix; percentage of days removed from ΔXNO_2_/ΔXCO; surface measurement details and column-surface correlations; mean daily absolute inequalities, inequalities in the frequency of days above the median and seventieth percentiles, with winds and temperature, and seasonally; NEI20 sector-level E_NO2_/E_CO_; differences in ΔXNO_2_/ΔXCO on weekends and weekdays; census tract-scale averaging kernels and city-level averaging kernel profiles. (PDF)

## References

[1] Clark L. P., Millet D. B., Marshall J. D. (2014). National Patterns in Environmental
Injustice and Inequality: Outdoor NO_2_ Air Pollution in
the United States. PLoS One.

[2] Tessum C. W., Paolella D. A., Chambliss S. E., Apte J. S., Hill J. D., Marshall J. D. (2021). PM_2.5_ polluters disproportionately and systemically
affect people of color in the United States. Sci. Adv..

[3] Wang Y., Liu P., Schwartz J., Castro E., Wang W., Chang H., Scovronick N., Shi L. (2023). Disparities in ambient nitrogen dioxide
pollution in the United States. Proc. Natl.
Acad. Sci. U. S. A..

[4] Demetillo M. A. G., Harkins C., McDonald B. C., Chodrow P. S., Sun K., Pusede S. E. (2021). Space-Based Observational Constraints on NO_2_ Air Pollution Inequality From Diesel Traffic in Major US Cities. Geophys. Res. Lett..

[5] Demetillo M. A. G., Navarro A., Knowles K. K., Fields K. P., Geddes J. A., Nowlan C. R., Janz S. J., Judd L. M., Al-Saadi J., Sun K., McDonald B. C., Diskin G. S., Pusede S. E. (2020). Observing Nitrogen
Dioxide Air Pollution Inequality Using High-Spatial-Resolution Remote
Sensing Measurements in Houston, Texas. Environ.
Sci. Technol..

[6] Dressel I. M., Demetillo M. A. G., Judd L. M., Janz S. J., Fields K. P., Sun K., Fiore A. M., McDonald B. C., Pusede S. E. (2022). Daily Satellite
Observations of Nitrogen Dioxide Air Pollution Inequality in New York
City, New York and Newark, New Jersey: Evaluation and Application. Environ. Sci. Technol..

[7] Clark L. P., Millet D. B., Marshall J. D. (2017). Changes
in Transportation-Related
Air Pollution Exposures by Race-Ethnicity and Socioeconomic Status:
Outdoor Nitrogen Dioxide in the United States in 2000 and 2010. Environ. Health Perspect..

[8] Liu J., Clark L. P., Bechle M. J., Hajat A., Kim S. Y., Robinson A. L., Sheppard L., Szpiro A. A., Marshall J. D. (2021). Disparities
in Air Pollution Exposure in the United States by Race/Ethnicity and
Income, 1990–2010. Environ. Health Perspect..

[9] Ofodile J., Pfannerstill E. Y., Arata C., Pusede S. E., Ivey C. E., Goldstein A. H. (2025). Inequality
in Hazardous Air Pollutant Emissions and
Concentrations Measured Over Los Angeles. Environ.
Sci. Technol..

[10] McDonald B. C., Gentner D. R., Goldstein A. H., Harley R. A. (2013). Long-Term Trends
in Motor Vehicle Emissions in U.S. Urban Areas. Environ. Sci. Technol..

[11] Reşitoğlu İ. A., Altinişik K., Keskin A. (2015). The pollutant emissions
from diesel-engine vehicles and exhaust aftertreatment systems. Clean Technol. Environ. Policy.

[12] Lama S., Houweling S., Boersma K. F., Eskes H., Aben I., Denier van
der Gon H. A. C., Krol M. C., Dolman H., Borsdorff T., Lorente A. (2020). Quantifying burning efficiency in
megacities using the NO_2_/CO ratio from the Tropospheric
Monitoring Instrument (TROPOMI). Atmos. Chem.
Phys..

[13] MacDonald C. G., Mastrogiacomo J. P., Laughner J. L., Hedelius J. K., Nassar R., Wunch D. (2023). Estimating enhancement ratios of
nitrogen dioxide, carbon monoxide
and carbon dioxide using satellite observations. Atmos. Chem. Phys..

[14] Simon H., Valin L. C., Baker K. R., Henderson B. H., Crawford J. H., Pusede S. E., Kelly J. T., Foley K. M., Chris Owen R., Cohen R. C., Timin B., Weinheimer A. J., Possiel N., Misenis C., Diskin G. S., Fried A. (2018). Characterizing
CO and NO_y_ Sources and Relative Ambient Ratios in the Baltimore
Area Using Ambient Measurements and Source Attribution Modeling. J. Geophys. Res.: Atmos..

[15] Anderson D. C., Loughner C. P., Diskin G., Weinheimer A., Canty T. P., Salawitch R. J., Worden H. M., Fried A., Mikoviny T., Wisthaler A., Dickerson R. R. (2014). Measured
and modeled CO and NO_y_ in DISCOVER-AQ: An evaluation of
emissions and chemistry over the eastern US. Atmos. Environ..

[16] Su J. G., Jerrett M., Morello-Frosch R., Jesdale B. M., Kyle A. D. (2012). Inequalities
in cumulative environmental burdens among three urbanized counties
in California. Environ. Int..

[17] Raub J. A. (1999). Health
effects of exposure to ambient carbon monoxide. Chemosphere Glob. Change Sci..

[18] Townsend C. L., Maynard R. L. (2002). Effects on health
of prolonged exposure to low concentrations
of carbon monoxide. Occup. Environ. Med..

[19] Atkinson R. W., Butland B. K., Anderson H. R., Maynard R. L. (2018). Long-term Concentrations
of Nitrogen Dioxide and Mortality: A Meta-analysis of Cohort Studies. Epidemiology.

[20] Anenberg S. C., Henze D. K., Tinney V., Kinney P. L., Raich W., Fann N., Malley C. S., Roman H., Lamsal L., Duncan B., Martin R. V., van Donkelaar A., Brauer M., Doherty R., Jonson J. E., Davila Y., Sudo K., Kuylenstierna J. C. I. (2018). Estimates of the Global Burden of
Ambient PM_2.5_, Ozone, and NO_2_ on Asthma Incidence
and Emergency Room Visits. Environ. Health Perspect..

[21] Shah V., Jacob D. J., Li K., Silvern R. F., Zhai S., Liu M., Lin J., Zhang Q. (2020). Effect of changing NO_x_ lifetime on the seasonality and long-term trends of satellite-observed
tropospheric NO_2_ columns over China. Atmos. Chem. Phys..

[22] Lange K., Richter A., Burrows J. P. (2022). Variability
of nitrogen oxide emission
fluxes and lifetimes estimated from Sentinel-5P TROPOMI observations. Atmos. Chem. Phys..

[23] National Emissions Inventory (NEI) Data. U.S. Environmental Protection Agency, 2020.

[24] Karner A.
A., Eisinger D. S., Niemeier D. A. (2010). Near-Roadway Air Quality: Synthesizing
the Findings from Real-World Data. Environ.
Sci. Technol..

[25] Apte J. S., Messier K. P., Gani S., Brauer M., Kirchstetter T. W., Lunden M. M., Marshall J. D., Portier C. J., Vermeulen R. C. H., Hamburg S. P. (2017). High-Resolution Air Pollution Mapping with Google Street
View Cars: Exploiting Big Data. Environ. Sci.
Technol..

[26] Gonzalez A., Millet D. B., Yu X., Wells K. C., Griffis T. J., Baier B. C., Campbell P. C., Choi Y., DiGangi J. P., Gvakharia A., Halliday H. S., Kort E. A., McKain K., Nowak J. B., Plant G. (2021). Fossil Versus Nonfossil CO Sources
in the US: New Airborne Constraints From ACT-America and GEM. Geophys. Res. Lett..

[27] Parrish D. D., Trainer M., Hereid D., Williams E. J., Olszyna K. J., Harley R. A., Meagher J. F., Fehsenfeld F. C. (2002). Decadal
change in carbon monoxide to nitrogen oxide ratio in U.S. vehicular
emissions. J. Geophys. Res.: Atmos..

[28] Hassler B., McDonald B. C., Frost G. J., Borbon A., Carslaw D. C., Civerolo K., Granier C., Monks P. S., Monks S., Parrish D. D., Pollack I. B., Rosenlof K. H., Ryerson T. B., von Schneidemesser E., Trainer M. (2016). Analysis of long-term observations
of NO_x_ and CO in megacities and application to constraining
emissions inventories. Geophys. Res. Lett..

[29] McDonald B. C., McBride Z. C., Martin E. W., Harley R. A. (2014). High-resolution
mapping of motor vehicle carbon dioxide emissions. J. Geophys. Res.: Atmos..

[30] Hakkarainen J., Ialongo I., Tamminen J. (2016). Direct space-based
observations of
anthropogenic CO_2_ emission areas from OCO-2. Geophys. Res. Lett..

[31] Konovalov I. B., Berezin E. V., Ciais P., Broquet G., Beekmann M., Hadji-Lazaro J., Clerbaux C., Andreae M. O., Kaiser J. W., Schulze E. D. (2014). Constraining CO_2_ emissions from open biomass
burning by satellite observations of co-emitted species: a method
and its application to wildfires in Siberia. Atmos. Chem. Phys..

[32] Konovalov I. B., Berezin E. V., Ciais P., Broquet G., Zhuravlev R. V., Janssens-Maenhout G. (2016). Estimation
of fossil-fuel CO_2_ emissions
using satellite measurements of “proxy” species. Atmos. Chem. Phys..

[33] Reuter M., Buchwitz M., Schneising O., Krautwurst S., O’Dell C. W., Richter A., Bovensmann H., Burrows J. P. (2019). Towards monitoring localized CO_2_ emissions
from space: co-located regional CO_2_ and NO_2_ enhancements
observed by the OCO-2 and S5P satellites. Atmos.
Chem. Phys..

[34] Silva S. J., Arellano A. F. (2017). Characterizing Regional-Scale Combustion
Using Satellite
Retrievals of CO, NO_2_ and CO_2_. Remote Sens..

[35] Silva S. J., Arellano A. F., Worden H. M. (2013). Toward anthropogenic combustion emission
constraints from space-based analysis of urban CO_2_/CO sensitivity. Geophys. Res. Lett..

[36] Miyazaki K., Eskes H., Sudo K., Boersma K. F., Bowman K., Kanaya Y. (2017). Decadal changes in
global surface NO_x_ emissions
from multi-constituent satellite data assimilation. Atmos. Chem. Phys..

[37] Fujita E. M., Croes B. E., Bennett C. L., Lawson D. R., Lurmann F. W., Main H. H. (1992). Comparison of Emission
Inventory and Ambient Concentration
Ratios of CO, NMOG, and NO_x_ in California’s South
Coast Air Basin. J. Air Waste Manage. Assoc..

[38] Salmon O. E., Shepson P. B., Ren X., He H., Hall D. L., Dickerson R. R., Stirm B. H., Brown S. S., Fibiger D. L., McDuffie E. E., Campos T. L., Gurney K. R., Thornton J. A. (2018). Top-Down
Estimates of NO and CO Emissions From Washington, D.C.-Baltimore During
the WINTER Campaign. J. Geophys. Res.: Atmos..

[39] Anderson D. C., Lindsay A., DeCarlo P. F., Wood E. C. (2021). Urban Emissions
of Nitrogen Oxides, Carbon Monoxide, and Methane Determined from Ground-Based
Measurements in Philadelphia. Environ. Sci.
Technol..

[40] Dressel I. M., Zhang S., Demetillo M. A. G., Yu S., Fields K., Judd L. M., Nowlan C. R., Sun K., Kotsakis A., Turner A. J., Pusede S. E. (2024). Neighborhood-Level Nitrogen Dioxide
Inequalities Contribute to Surface Ozone Variability in Houston. ACS ES&T Air.

[41] Landgraf J., aan de Brugh J., Scheepmaker R., Borsdorff T., Hu H., Houweling S., Butz A., Aben I., Hasekamp O. (2016). Carbon monoxide
total column retrievals from TROPOMI shortwave infrared measurements. Atmos. Meas. Tech..

[42] Landgraf, J. ; Borsdorff, T. ; Langerock, B. ; Keppens, A. S5P Mission Performance Centre Carbon Monoxide [L2__CO:]; SRON Netherlands Institute for Space Research, Utrecht, 2023.

[43] Martínez-Alonso S., Deeter M., Worden H., Borsdorff T., Aben I., Commane R., Daube B., Francis G., George M., Landgraf J., Mao D., McKain K., Wofsy S. (2020). 1.5 years of TROPOMI CO measurements:
comparisons to MOPITT and ATom. Atmos. Meas.
Tech..

[44] Borsdorff T., Aan de Brugh J., Hu H., Aben I., Hasekamp O., Landgraf J. (2018). Measuring Carbon Monoxide With TROPOMI:
First Results
and a Comparison With ECMWF-IFS Analysis Data. Geophys. Res. Lett..

[45] Boersma K. F., Eskes H. J., Dirksen R. J., van der A R. J., Veefkind J. P., Stammes P., Huijnen V., Kleipool Q. L., Sneep M., Claas J., Leitão J., Richter A., Zhou Y., Brunner D. (2011). An improved tropospheric
NO_2_ column retrieval algorithm for the Ozone Monitoring
Instrument. Atmos. Meas. Tech..

[46] Boersma K. F., Eskes H. J., Richter A., De Smedt I., Lorente A., Beirle S., van Geffen J. H. G. M., Zara M., Peters E., Van Roozendael M., Wagner T., Maasakkers J. D., van der A R. J., Nightingale J., De Rudder A., Irie H., Pinardi G., Lambert J. C., Compernolle S. C. (2018). Improving
algorithms and uncertainty estimates for satellite NO_2_ retrievals:
results from the quality assurance for the essential climate variables
(QA4ECV) project. Atmos. Meas. Tech..

[47] Lorente A., Folkert Boersma K., Yu H., Dörner S., Hilboll A., Richter A., Liu M., Lamsal L. N., Barkley M., De Smedt I., Van Roozendael M., Wang Y., Wagner T., Beirle S., Lin J. T., Krotkov N., Stammes P., Wang P., Eskes H. J., Krol M. (2017). Structural uncertainty in air mass factor calculation for NO_2_ and HCHO satellite retrievals. Atmos.
Meas. Tech..

[48] van
Geffen J. H. G. M., Boersma K. F., Van Roozendael M., Hendrick F., Mahieu E., De Smedt I., Sneep M., Veefkind J. P. (2015). Improved spectral fitting of nitrogen dioxide from
OMI in the 405–465 nm window. Atmos.
Meas. Tech..

[49] Zara M., Boersma K. F., De Smedt I., Richter A., Peters E., van Geffen J. H. G. M., Beirle S., Wagner T., Van Roozendael M., Marchenko S., Lamsal L. N., Eskes H. J. (2018). Improved slant column
density retrieval of nitrogen dioxide and formaldehyde for OMI and
GOME-2A from QA4ECV: intercomparison, uncertainty characterisation,
and trends. Atmos. Meas. Tech..

[50] Eskes, H. J. ; Eichmann, K.-U. S5P Mission Performance Centre Nitrogen Dioxide [L2__NO2___]; Readme, 2023.

[51] Dressel I.
M., Zhang S., Demetillo M. A. G., Yu S., Fields K., Judd L. M., Nowlan C. R., Sun K., Kotsakis A., Turner A. J., Pusede S. E. (2024). Neighborhood-level nitrogen dioxide
inequalities contribute to surface ozone variability in Houston, Texas. ACS ES&T Air.

[52] Wunch D., Toon G. C., Hedelius J. K., Vizenor N., Roehl C. M., Saad K. M., Blavier J. F. L., Blake D. R., Wennberg P. O. (2016). Quantifying
the loss of processed natural gas within California’s South
Coast Air Basin using long-term measurements of ethane and methane. Atmos. Chem. Phys..

[53] Epps A., Dressel I. M., Guo X., Odanibe M., Fields K. P., Carlton A. M. G., Sun K., Pusede S. E. (2025). Satellite Observations
of Atmospheric Ammonia Inequalities Associated with Industrialized
Swine Facilities in Eastern North Carolina. Environ. Sci. Technol..

[54] Kerr G. H., Goldberg D. L., Anenberg S. C. (2021). COVID-19
pandemic reveals persistent
disparities in nitrogen dioxide pollution. Proc.
Natl. Acad. Sci. U. S. A..

[55] Kroll J. H., Heald C. L., Cappa C. D., Farmer D. K., Fry J. L., Murphy J. G., Steiner A. L. (2020). The complex chemical effects of COVID-19
shutdowns on air quality. Nat. Chem..

[56] Fisher B. L., Lamsal L. N., Fasnacht Z., Oman L. D., Joiner J., Krotkov N. A., Choi S., Qin W., Yang E.-S. (2024). Revised
estimates of NO_2_ reductions during the COVID-19 lockdowns
using updated TROPOMI NO_2_ retrievals and model simulations. Atmos. Environ..

[57] Manson, S. ; Schroeder, J. , Van Riper, D. ; Knowles, K. ; Kugler, T. ; Roberts, F. ; Ruggles, S. IPUMS National Historical Geographic Information System: Version 18.0 [dataset]; IPUMS: Minneapolis, MN, 2023.

[58] Spielman S.
E., Folch D., Nagle N. (2014). Patterns and causes of uncertainty
in the American Community Survey. Appl. Geogr..

[59] U. S. Census Bureau Understanding and Using American Community Survey Data: What All Data Users Need to Know; U. S. Census Bureau: Washington, DC, 2020.

[60] Morckel V. (2025). Population
decline and data discrepancies: evaluating ACS estimates and comprehensive
plan projections for a subset of U.S. shrinking cities. Pla. Pract. Res..

[61] Trujillo-Ortiz, A. gmregress; MATLAB Central File Exchange 2024

[62] Levy I., Mihele C., Lu G., Narayan J., Brook J. R. (2014). Evaluating
Multipollutant Exposure and Urban Air Quality: Pollutant Interrelationships,
Neighborhood Variability, and Nitrogen Dioxide as a Proxy Pollutant. Environ. Health Perspect..

[63] Air Data: Air Quality Data Collected at Outdoor Monitors Across the U.S; U.S. Environmental Protection Agency 2024

[64] Winer A. M., Peters J. W., Smith J. P., Pitts J. N. (1974). Response of commercial chemiluminescent
nitric oxide-nitrogen dioxide
analyzers to other nitrogen-containing compounds. Environ. Sci. Technol..

[65] Dunlea E. J., Herndon S. C., Nelson D. D., Volkamer R. M., San Martini F., Sheehy P. M., Zahniser M. S., Shorter J. H., Wormhoudt J. C., Lamb B. K., Allwine E. J., Gaffney J. S., Marley N. A., Grutter M., Marquez C., Blanco S., Cardenas B., Retama A., Ramos Villegas C. R., Kolb C. E., Molina L. T., Molina M. J. (2007). Evaluation of nitrogen
dioxide chemiluminescence monitors
in a polluted urban environment. Atmos. Chem.
Phys..

[66] Steinbacher M., Zellweger C., Schwarzenbach B., Bugmann S., Buchmann B., Ordóñez C., Prevot A. S. H., Hueglin C. (2007). Nitrogen oxide
measurements at rural sites in Switzerland: Bias of conventional measurement
techniques. J. Geophys. Res: Atmos..

[67] Russell A. R., Valin L. C., Bucsela E. J., Wenig M. O., Cohen R. C. (2010). Space-based
Constraints on Spatial and Temporal Patterns of NO_x_ Emissions
in California, 2005–2008. Environ. Sci.
Technol..

[68] ASOS Network , ASOS-AWOS-METAR Data Download; Iowa State University, https://mesonet.agron.iastate.edu/request/download.phtml (last access: June 4, 2026).

[69] Beirle S., Borger C., Dörner S., Li A., Hu Z., Liu F., Wang Y., Wagner T. (2019). Pinpointing nitrogen oxide emissions
from space. Sci. Adv..

[70] de
Foy B., Lu Z., Streets D. G., Lamsal L. N., Duncan B. N. (2015). Estimates
of power plant NO_x_ emissions and lifetimes from OMI NO_2_ satellite retrievals. Atmos. Environ..

[71] McDonald B. C., McKeen S. A., Cui Y. Y., Ahmadov R., Kim S.-W., Frost G. J., Pollack I. B., Peischl J., Ryerson T. B., Holloway J. S., Graus M., Warneke C., Gilman J. B., de Gouw J. A., Kaiser J., Keutsch F. N., Hanisco T. F., Wolfe G. M., Trainer M. (2018). Modeling Ozone
in the Eastern U.S.
using a Fuel-Based Mobile Source Emissions Inventory. Environ. Sci. Technol..

[72] Harkins C., McDonald B. C., Henze D. K., Wiedinmyer C. (2021). A fuel-based
method for updating mobile source emissions during the COVID-19 pandemic. Environ. Res. Lett..

[73] McDonald B. C., Dallmann T. R., Martin E. W., Harley R. A. (2012). Long-term trends
in nitrogen oxide emissions from motor vehicles at national, state,
and air basin scales. J. Geophys. Res.: Atmos..

[74] Boersma K. F., Eskes H. J., Brinksma E. J. (2004). Error analysis
for tropospheric NO_2_ retrieval from space. J. Geophys. Res.:
Atmos..

[75] Wing O. E. J., Lehman W., Bates P. D., Sampson C. C., Quinn N., Smith A. M., Neal J. C., Porter J. R., Kousky C. (2022). Inequitable
patterns of US flood risk in the Anthropocene. Nat. Clim. Change.

[76] Bakkensen L. A., Ma L. (2020). Sorting over flood risk and implications
for policy reform. J. Environ. Econ. Manag..

[77] Jarvis, A. ; Guevara, E. ; Reuter, H. I. ; Nelson, A. NASA Shuttle Radar Topographic Mission (SRTM) 90-m Digital Elevation Database Version 4 2018.

[78] Zuraski K., Harkins C., Peischl J., Coggon M. M., Stockwell C. E., Robinson M. A., Gilman J., Warneke C., McDonald B. C., Brown S. S. (2025). On-Road Measurements
of Nitrogen Oxides, CO, CO2, and
VOC Emissions in Two Southwestern U.S. Cities. ACS ES&T Air.

[79] Marais E. A., Jacob D. J., Choi S., Joiner J., Belmonte-Rivas M., Cohen R. C., Beirle S., Murray L. T., Schiferl L. D., Shah V., Jaeglé L. (2018). Nitrogen oxides
in the global upper
troposphere: interpreting cloud-sliced NO_2_ observations
from the OMI satellite instrument. Atmos. Chem.
Phys..

[80] Shah V., Jacob D. J., Dang R., Lamsal L. N., Strode S. A., Steenrod S. D., Boersma K. F., Eastham S. D., Fritz T. M., Thompson C., Peischl J., Bourgeois I., Pollack I. B., Nault B. A., Cohen R. C., Campuzano-Jost P., Jimenez J. L., Andersen S. T., Carpenter L. J., Sherwen T., Evans M. J. (2023). Nitrogen oxides in the free troposphere:
implications for tropospheric oxidants and the interpretation of satellite
NO_2_ measurements. Atmos. Chem. Phys..

[81] Travis K. R., Jacob D. J., Fisher J. A., Kim P. S., Marais E. A., Zhu L., Yu K., Miller C. C., Yantosca R. M., Sulprizio M. P., Thompson A. M., Wennberg P. O., Crounse J. D., St. Clair J. M., Cohen R. C., Laughner J. L., Dibb J. E., Hall S. R., Ullmann K., Wolfe G. M., Pollack I. B., Peischl J., Neuman J. A., Zhou X. (2016). Why do models overestimate surface
ozone in the Southeast United States?. Atmos.
Chem. Phys..

[82] Dang R., Jacob D. J., Shah V., Eastham S. D., Fritz T. M., Mickley L. J., Liu T., Wang Y., Wang J. (2023). Background
nitrogen dioxide (NO2) over the United States and its implications
for satellite observations and trends: effects of nitrate photolysis,
aircraft, and open fires. Atmos. Chem. Phys..

[83] Qu Z., Jacob D. J., Silvern R. F., Shah V., Campbell P. C., Valin L. C., Murray L. T. (2021). US COVID-19 Shutdown Demonstrates
Importance of Background NO_2_ in Inferring NO_x_ Emissions From Satellite NO_2_ Observations. Geophys. Res. Lett..

[84] Silvern R. F., Jacob D. J., Mickley L. J., Sulprizio M. P., Travis K. R., Marais E. A., Cohen R. C., Laughner J. L., Choi S., Joiner J., Lamsal L. N. (2019). Using satellite
observations of tropospheric NO_2_ columns to infer long-term
trends in US NO_x_ emissions: the importance of accounting
for the free tropospheric NO_2_ background. Atmos. Chem. Phys..

[85] Singh H. B., Salas L., Herlth D., Kolyer R., Czech E., Avery M., Crawford J. H., Pierce R. B., Sachse G. W., Blake D. R., Cohen R. C., Bertram T. H., Perring A., Wooldridge P. J., Dibb J., Huey G., Hudman R. C., Turquety S., Emmons L. K., Flocke F., Tang Y., Carmichael G. R., Horowitz L. W. (2007). Reactive nitrogen distribution and
partitioning in the North American troposphere and lowermost stratosphere. J. Geophys. Res.: Atmos..

[86] Jiang Z., Zhu R., Miyazaki K., McDonald B. C., Klimont Z., Zheng B., Boersma K. F., Zhang Q., Worden H., Worden J. R., Henze D. K., Jones D. B. A., Denier van der Gon H. A. C., Eskes H. (2022). Decadal Variabilities in Tropospheric Nitrogen Oxides
Over United States, Europe, and China. J. Geophys.
Res.: Atmos..

[87] Zhu Q., Laughner J. L., Cohen R. C. (2019). Lightning NO_2_ simulation
over the contiguous US and its effects on satellite NO_2_ retrievals. Atmos. Chem. Phys..

[88] Jiang Z., McDonald B. C., Worden H., Worden J. R., Miyazaki K., Qu Z., Henze D. K., Jones D. B. A., Arellano A. F., Fischer E. V., Zhu L., Boersma K. F. (2018). Unexpected slowdown of US pollutant emission reduction
in the past decade. Proc. Natl. Acad. Sci. U.
S. A..

[89] Zhang Y., Wang Y., Chen G., Smeltzer C., Crawford J., Olson J., Szykman J., Weinheimer A. J., Knapp D. J., Montzka D. D., Wisthaler A., Mikoviny T., Fried A., Diskin G. (2016). Large vertical gradient
of reactive nitrogen oxides in the boundary layer: Modeling analysis
of DISCOVER-AQ 2011 observations. J. Geophys.
Res.: Atmos..

[90] Marr L. C., Harley R. A. (2002). Spectral analysis of weekday–weekend differences
in ambient ozone, nitrogen oxide, and non-methane hydrocarbon time
series in California. Atmos. Environ..

[91] Marr L. C., Harley R. A. (2002). Modeling the Effect of Weekday–Weekend Differences
in Motor Vehicle Emissions on Photochemical Air Pollution in Central
California. Environ. Sci. Technol..

[92] Pusede S.
E., Cohen R. C. (2012). On the
observed response of ozone to NO_x_ and VOC reactivity reductions
in San Joaquin Valley California 1995–present. Atmos. Chem. Phys..

[93] Thornton J. A., Wooldridge P. J., Cohen R. C., Martinez M., Harder H., Brune W. H., Williams E. J., Roberts J. M., Fehsenfeld F. C., Hall S. R., Shetter R. E., Wert B. P., Fried A. (2002). Ozone production
rates as a function of NO_x_ abundances and HO_x_ production rates in the Nashville urban plume. J. Geophys. Res.: Atmos..

[94] Pollack I. B., Ryerson T. B., Trainer M., Parrish D. D., Andrews A. E., Atlas E. L., Blake D. R., Brown S. S., Commane R., Daube B. C., de Gouw J. A., Dubé W. P., Flynn J., Frost G. J., Gilman J. B., Grossberg N., Holloway J. S., Kofler J., Kort E. A., Kuster W. C., Lang P. M., Lefer B., Lueb R. A., Neuman J. A., Nowak J. B., Novelli P. C., Peischl J., Perring A. E., Roberts J. M., Santoni G., Schwarz J. P., Spackman J. R., Wagner N. L., Warneke C., Washenfelder R. A., Wofsy S. C., Xiang B. (2012). Airborne and ground-based observations
of a weekend effect in ozone, precursors, and oxidation products in
the California South Coast Air Basin. J. Geophys.
Res.: Atmos..

[95] Harley R. A., Marr L. C., Lehner J. K., Giddings S. N. (2005). Changes in Motor
Vehicle Emissions on Diurnal to Decadal Time Scales and Effects on
Atmospheric Composition. Environ. Sci. Technol..

[96] Chambliss S. E., Pinon C. P. R., Messier K. P., LaFranchi B., Upperman C. R., Lunden M. M., Robinson A. L., Marshall J. D., Apte J. S. (2021). Local- and regional-scale
racial and ethnic disparities
in air pollution determined by long-term mobile monitoring. Proc. Natl. Acad. Sci. U. S. A..

[97] Schiferl L. D., Cao C., Dalton B., Hallward-Driemeier A., Toledo-Crow R., Commane R. (2024). Multi-year observations of variable
incomplete combustion
in the New York megacity. Atmos. Chem. Phys..

[98] Washenfelder R. A., Trainer M., Frost G. J., Ryerson T. B., Atlas E. L., de Gouw J. A., Flocke F. M., Fried A., Holloway J. S., Parrish D. D., Peischl J., Richter D., Schauffler S. M., Walega J. G., Warneke C., Weibring P., Zheng W. (2010). Characterization
of NO, SO_2_, ethene, and propene from industrial emission
sources in Houston, Texas. J. Geophys. Res.:
Atmos..

[99] Wallace H. W., Jobson B. T., Erickson M. H., McCoskey J. K., VanReken T. M., Lamb B. K., Vaughan J. K., Hardy R. J., Cole J. L., Strachan S. M., Zhang W. (2012). Comparison
of wintertime CO to NO_x_ ratios to MOVES and MOBILE6.2 on-road
emissions inventories. Atmos. Environ..

[100] Anderson L. D., Dix B., Schnell J., Yokelson R., Veefkind J. P., Ahmadov R., de Gouw J. (2023). Analyzing
the Impact
of Evolving Combustion Conditions on the Composition of Wildfire Emissions
Using Satellite Data. Geophys. Res. Lett..

[101] Jaeglé L., Shah V., Thornton J. A., Lopez-Hilfiker F. D., Lee B. H., McDuffie E. E., Fibiger D., Brown S. S., Veres P., Sparks T. L., Ebben C. J., Wooldridge P. J., Kenagy H. S., Cohen R. C., Weinheimer A. J., Campos T. L., Montzka D. D., Digangi J. P., Wolfe G. M., Hanisco T., Schroder J. C., Campuzano-Jost P., Day D. A., Jimenez J. L., Sullivan A. P., Guo H., Weber R. J. (2018). Nitrogen Oxides Emissions, Chemistry, Deposition, and
Export Over the Northeast United States During the WINTER Aircraft
Campaign. J. Geophys. Res.: Atmos..

[102] Saha P. K., Khlystov A., Snyder M. G., Grieshop A. P. (2018). Characterization
of air pollutant concentrations, fleet emission factors, and dispersion
near a North Carolina interstate freeway across two seasons. Atmos. Environ..

[103] He H., Hembeck L., Hosley K. M., Canty T. P., Salawitch R. J., Dickerson R. R. (2013). High ozone
concentrations on hot days: The role of
electric power demand and NO_x_ emissions. Geophys. Res. Lett..

[104] Demetillo M. A. G., Judd L. M., Travis K. R., Crawford J. H., Rawat P., Hair J. W., Fenn M., Ferrare R., Shingler T., Sullivan J. T., Walter P., Flynn J., Griggs T. (2025). Observing Lower-Tropospheric Ozone
Spatiotemporal Variability
With Airborne Lidar and Surface Monitors in Houston, Texas. J. Geophys. Res.: Atmos..

[105] Meier S., Koene E. F. M., Krol M., Brunner D., Damm A., Kuhlmann G. (2024). A lightweight NO_2_-to-NO_x_ conversion model for quantifying NOx emissions of point sources
from NO_2_ satellite observations. Atmos. Chem. Phys..

[106] Miller S. M., Matross D. M., Andrews A. E., Millet D. B., Longo M., Gottlieb E. W., Hirsch A. I., Gerbig C., Lin J. C., Daube B. C., Hudman R. C., Dias P. L. S., Chow V. Y., Wofsy S. C. (2008). Sources of carbon
monoxide and formaldehyde
in North America determined from high-resolution atmospheric data. Atmos. Chem. Phys..

[107] Brioude J., Angevine W. M., Ahmadov R., Kim S. W., Evan S., McKeen S. A., Hsie E. Y., Frost G. J., Neuman J. A., Pollack I. B., Peischl J., Ryerson T. B., Holloway J., Brown S. S., Nowak J. B., Roberts J. M., Wofsy S. C., Santoni G. W., Oda T., Trainer M. (2013). Top-down estimate
of surface flux in the Los Angeles Basin using a mesoscale inverse
modeling technique: assessing anthropogenic emissions of CO, NO_x_ and CO_2_ and their impacts. Atmos. Chem. Phys..

[108] Castellanos P., Marufu L. T., Doddridge B. G., Taubman B. F., Schwab J. J., Hains J. C., Ehrman S. H., Dickerson R. R. (2011). Ozone, oxides of nitrogen, and carbon monoxide during
pollution events over the eastern United States: An evaluation of
emissions and vertical mixing. J. Geophys. Res.:
Atmos..

[109] Lopez-Coto I., Ren X., Karion A., McKain K., Sweeney C., Dickerson R. R., McDonald B. C., Ahn D. Y., Salawitch R. J., He H., Shepson P. B., Whetstone J. R. (2022). Carbon
Monoxide Emissions from the Washington, DC, and Baltimore Metropolitan
Area: Recent Trend and COVID-19 Anomaly. Environ.
Sci. Technol..

[110] Marr L. C., Moore T. O., Klapmeyer M. E., Killar M. B. (2013). Comparison of NOx Fluxes Measured by Eddy Covariance
to Emission Inventories and Land Use. Environ.
Sci. Technol..

[111] Canty T. P., Hembeck L., Vinciguerra T. P., Anderson D. C., Goldberg D. L., Carpenter S. F., Allen D. J., Loughner C. P., Salawitch R. J., Dickerson R. R. (2015). Ozone and NO_x_ chemistry in the eastern US:
evaluation of CMAQ/CB05 with satellite (OMI) data. Atmos. Chem. Phys..

[112] Brunekreef B., Holgate S. T. (2002). Air pollution and
health. Lancet.

[113] Multi-Pollutant Emissions Standards for Model Years 2027 and Later Light-Duty and Medium-Duty Vehicles, 40 CFR Parts 85, 86, 600, 1036, 1037, 1066, and 1068, EPA-HQ-OAR-2022–0829; FRL-8953–04-OAR, RIN 2060-AV49; US EPA April 4, 2024.

