# Supplementary material for: TROPOMI CO and NO2 as Observational Constraints on the Sources of Air Pollution Inequalities in US Cities
Source: Environ Sci Technol. 2026 Jun 29;60(27):19397–412. doi: 10.1021/acs.est.5c07455 (PMC13374096; doi:10.1021/acs.est.5c07455)
Supplement: Supplementary file 1 [file es5c07455_si_001.pdf]

Supporting Information

TROPOMI CO and NO_2 as observational constraints on the sources of air pollution inequalities in U.S. cities

AUTHOR NAMES: Xuehui Guo[1]*, Jon-Paul Mastrogiacomo[2], Debra Wunch[2], Isabella Dressel[1], Madeline A. Miles[1], Ziqi Gao[1], Andrew Gallego[1], and Sally Pusede[1]*

AUTHOR ADDRESSES:

[1] Department of Environmental Sciences, University of Virginia, Charlottesville, Virginia 22904, United States

[2] Department of Physics, University of Toronto, Toronto, Ontario M5S 1A7, Canada

*Correspondence to: Xuehui Guo (sdh2nv@virginia.edu) and Sally E. Pusede (sepusede@virginia.edu)

SI includes: 41 pages, 9 figures, 3 appendices, 5 equations, and 18 tables

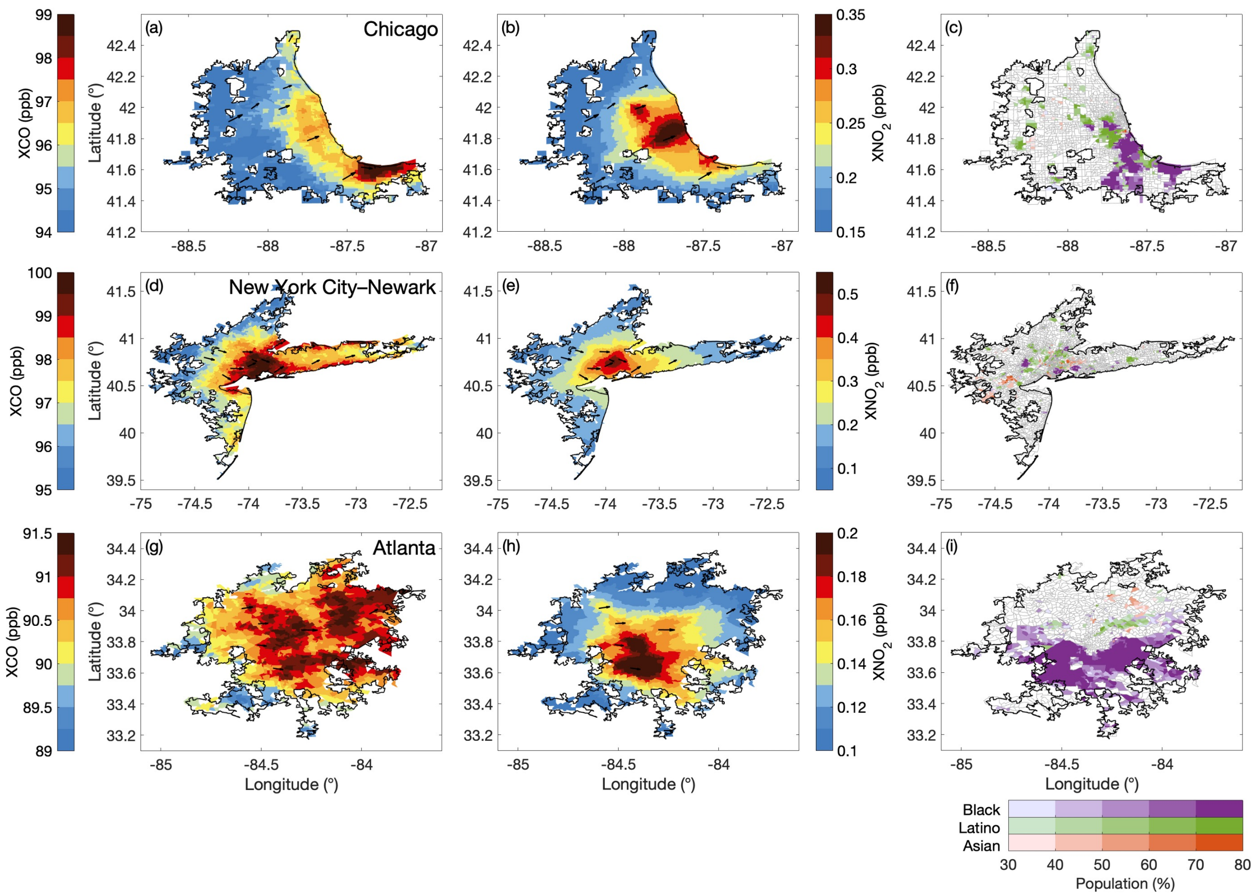

Figure S1. Mean daily census tract-scale XCO and XNO_2 over June 2018–February 2023 (excluding March 2020–February 2021) in Chicago (a–c), New York City–Newark (d–f), and Atlanta (g–i) with populations (%) of Black (purple), Latino (green), and Asian (orange) populations in tracts where they are the largest race-ethnicity group. Mean daytime (12–3 pm LT) surface wind fields (black vectors) over the same period are shown. Thick black outlines are UA boundaries.

Table S1. Sensitivity of mean daily XCO_{norm} inequalities (%) and absolute XCO inequalities (ppb) to CO pixels over water and along coasts over June 2018–February 2023 (excluding March 2020–February 2021) in New York City–Newark. Pixels are classified using the surface classification parameter in TROPOMI datafiles: case 1, all pixels are used; case 2, water pixels are removed; and case 3, water and coastal pixels are removed. Water pixels are defined as those more than 50% over water. Coastal pixels are defined as those over water but that contain land (coastline).

	**Case 1**	**Case 2**	**Case 3**
**Population Group**		**Inequalities (%)**	
Black	11 ± 1	11 ± 1	12 ± 1
Latino	8 ± 1	8 ± 1	10 ± 1
Asian	8 ± 1	8 ± 1	9 ± 1
		**Inequalities (ppb)**	
Black	0.8 ± 0.1	0.8 ± 0.1	1.0 ± 0.1
Latino	0.7 ± 0.1	0.7 ± 0.1	0.7 ± 0.1
Asian	0.6 ± 0.1	0.6 ± 0.1	0.7 ± 0.1

Appendix S1. TROPOMI census tract-scale NO_2 inequalities have been evaluated in detail in Houston and New York City–Newark using oversampled and mean daily NO_2 TVCDs.[1-3] This was first done by Demetillo et al.,[2] who compared census tract-scale inequalities in Houston in NO_2 TVCDs oversampled to 0.01° × 0.01° with NO_2 columns (250 m × 500 m) from the NASA Geostationary Coastal and Air Pollution Events (GEO-CAPE) Airborne Simulator (GCAS), an airborne push-broom spectrometer resolving length scales of atmospheric dispersion. Demetillo et al.[2] also showed that intraurban spatial patterns in NO_2 columns reflected those at the surface based on 144 aircraft in-situ NO_2 vertical profiles and TROPOMI TVCD-surface mixing ratio correlations analyzed as a function of their separation distance. Dressel et al.[3, 4] demonstrated that mean daily TROPOMI NO_2 TVCDs (without oversampling) captured most census tract-scale NO_2 inequalities relative to spatiotemporally-coincident GCAS and GeoTASO (Geostationary Trace gas and Aerosol Sensor Optimization) columns in New York City–Newark and Houston. In New York City–Newark during the NASA Long Island Sound Tropospheric Ozone Study (LISTOS), bivariate linear regression slopes for census tract-scale inequalities in TROPOMI NO_2 TVCDs (*y*-axis) and GCAS/GeoTASO NO_2 columns (250 m × 250 m) (*x*-axis) were 0.82–1.05 for relative and 0.76–0.96 for absolute NO_2 inequalities, with Pearson correlation coefficients (r) of 0.82–0.97.[3] In Houston during the NASA TRacking Aerosol Convection ExpeRiment–Air Quality study (TRACER-AQ), bivariate linear regression slopes for tract-scale inequalities in TROPOMI NO_2 TVCDs and GCAS NO_2 columns (250 m × 560 m) were 0.66–1.08 for relative and 0.56–0.77 for absolute inequalities (r = 0.70–0.91).[4] Dressel et al.[4] explored differences in absolute NO_2 inequalities based on mean daily and oversampled NO_2 TVCDs (0.01° × 0.01°) using natural variability in TROPOMI pixel configurations, comparing NO_2 inequalities within individual S-5P orbits and eliminating most pixel overlap, as required for oversampling. Daily mean absolute NO_2

inequalities were ~30% larger and, therefore, more accurate with respect to GCAS measurements, than inequalities in oversampled TVCDs, which the authors attributed to the smoothing of grid-scale features during oversampling.[4, 5] Daily observations span a wider range of NO_2 column densities than oversampled TVCDs, particularly in the high tail of the NO_2 distribution.[4]

Table S2. Mean daily XCO_{norm} inequalities (%) and absolute XCO inequalities (ppb) over June 2018–February 2023 (excluding March 2020–February 2021) based on two XCO products with different pressure inputs. Product 1 is retrieved using the TROPOMI NO_2 pressure field. Product 2 is retrieved using the TROPOMI CO pressure field.

Population Group	Product 1 Relative Inequalities (%)	Product 2 Relative Inequalities (%)	Product 1 Absolute Inequalities (ppb)	Product 2 Absolute Inequalities (ppb)
**Los Angeles–Riverside**				
Black	7 ± 1	7 ± 1	0.5 ± 0.1	0.5 ± 0.1
Latino	15 ± 1	14 ± 1	1.3 ± 0.1	1.2 ± 0.1
Asian	11 ± 1	10 ± 1	0.9 ± 0.1	0.9 ± 0.1
**Houston**				
Black	3 ± 2	2 ± 3	0.3 ± 0.1	0.3 ± 0.1
Latino	5 ± 2	5 ± 2	0.4 ± 0.1	0.4 ± 0.1
Asian	–5 ± 4	–7 ± 5	–0.1 ± 0.1	–0.1 ± 0.1
**Phoenix**				
Black	10 ± 1	8 ± 1	0.5 ± 0.1	0.4 ± 0.1
Latino	15 ± 1	13 ± 1	0.8 ± 0.1	0.7 ± 0.1
Asian	2 ± 0	2 ± 0	0.1 ± 0.1	0.1 ± 0.1
**Chicago**				
Black	10 ± 1	10 ± 1	0.7 ± 0.1	0.7 ± 0.1
Latino	7 ± 1	7 ± 1	0.5 ± 0.1	0.5 ± 0.1
Asian	1 ± 1	1 ± 1	0.1 ± 0.1	0.1 ± 0.1
**New York City–Newark**				
Black	11 ± 1	11 ± 1	0.8 ± 0.1	0.8 ± 0.1
Latino	8 ± 1	8 ± 1	0.7 ± 0.1	0.7 ± 0.1
Asian	8 ± 1	9 ± 1	0.6 ± 0.1	0.7 ± 0.1
**Atlanta**				
Black	1 ± 1	1 ± 1	0.0 ± 0.1	0.0 ± 0.1
Latino	3 ± 1	3 ± 1	0.2 ± 0.1	0.2 ± 0.1
Asian	4 ± 1	3 ± 1	0.3 ± 0.1	0.2 ± 0.1

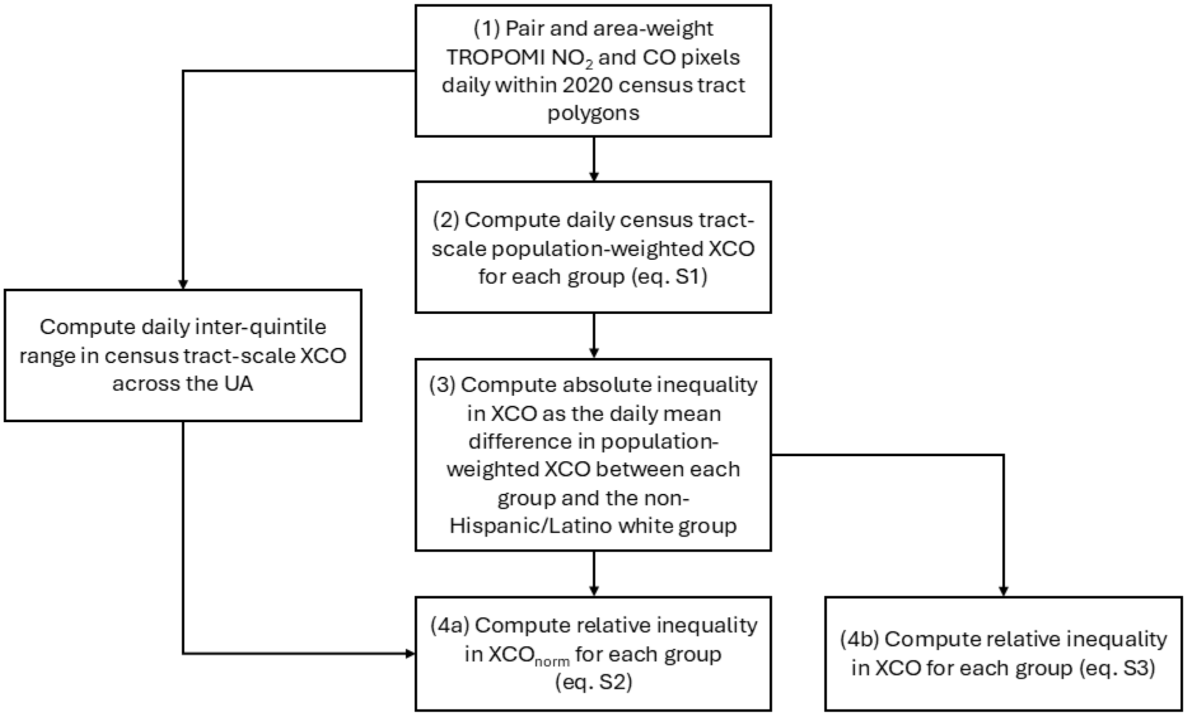

Figure S2. Workflow for computing population-weighted absolute inequalities (3), urban variability-normalized inequalities (4a), and relative inequalities as percent differences (4b) using XCO as an example. The process is repeated daily for XCO, CO VCDs, XNO_2, and NO_2 TVCDs.

Equation S1. The mean population-weighted value of k for group j, denoted $\overline{k_j}$, is calculated as the summation of the product of k in census tract i (k_i) and the population of group j in tract i ($p_{i,j}$) over all census tracts (n) divided by the summation of $p_{i,j}$ over all census tracts.

(Eq. S1) $$\overline{k}_j = \frac{\sum_{i=1}^{n} k_i \, p_{i,j}}{\sum_{i=1}^{n} p_{i,j}}$$

Equation S2. Urban variability-normalized inequality in *k* for race-ethnicity group *j*, defined as the absolute inequality in *k* for that group with respect to the non-Hispanic/Latino white group divided by the UA inter-quintile range multiplied by 100. Inter-quintile range is determined from all census tracts in the UA with observations on that day.

(Eq. S2) $$\text{Inequality} = 100 \times \frac{\bar{k}_{\text{j}} - \bar{k}_{\text{w}}}{k_{\text{80th-percentile}} - k_{\text{20th-percentile}}}$$

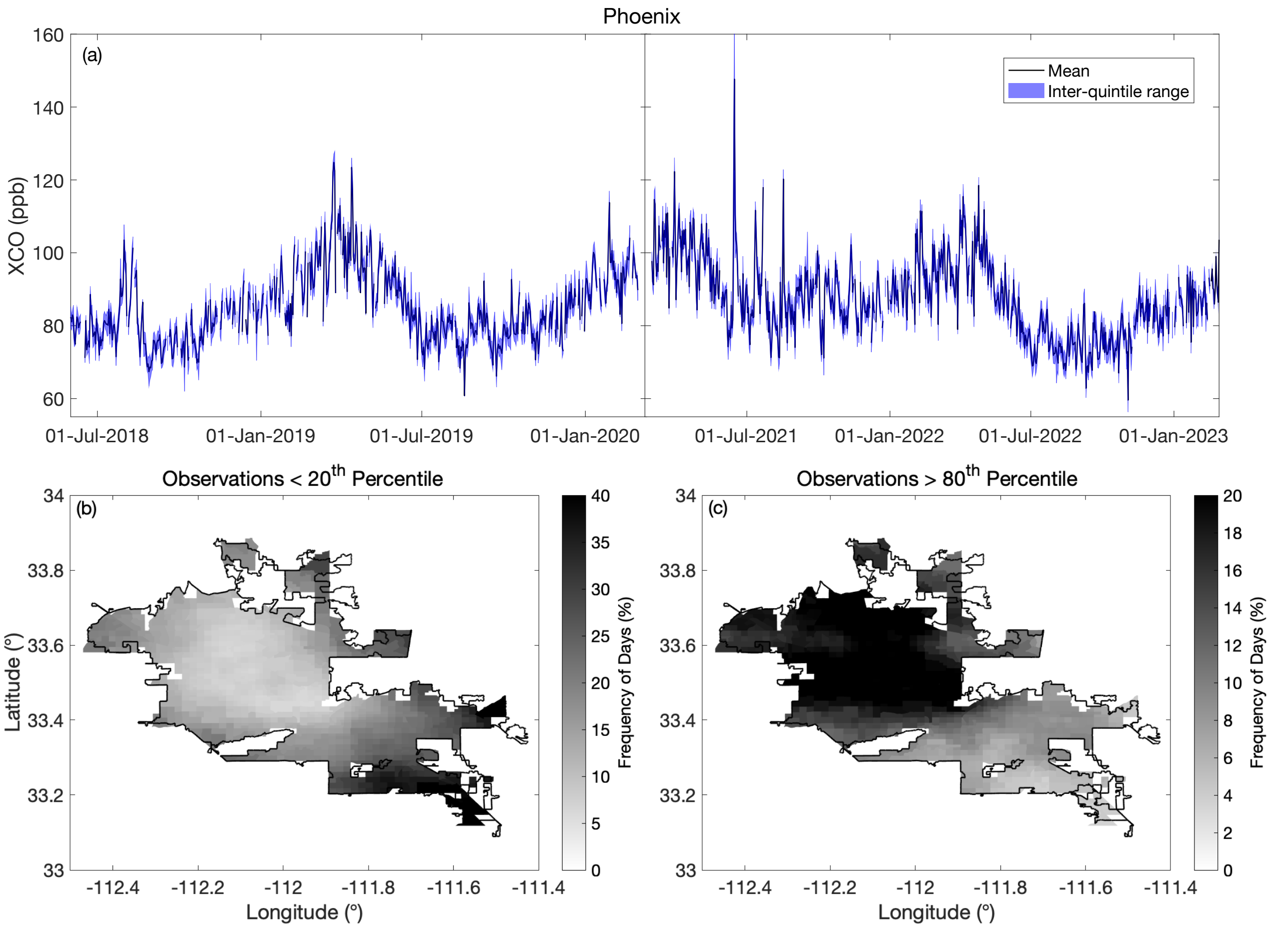

Figure S3. Timeseries of XCO in Phoenix over June 2018–February 2023 (excluding March 2020–February 2021, shown as the cutoff between panels), with the corresponding inter-quintile range used to compute inequalities in XCO_{norm} and $XNO_{2,norm}$ (a) and maps of the frequency of daily observations below the twentieth percentile (b) and above the eightieth percentile (c).

Equation S3. Relative inequality in k for race-ethnicity group j, defined as the percent difference in k between that population group with respect to the non-Hispanic/Latino white group.

(Eq. S3) $$\text{Inequality} = 100 \times \frac{\bar{k}_{\mathrm{j}} - \bar{k}_{\mathrm{w}}}{\frac{1}{2}(\bar{k}_{\mathrm{j}} + \bar{k}_{\mathrm{w}})}$$

Appendix S2. Mean daily TROPOMI census tract-scale inequality estimates are potentially affected by both the number of tracts across the UA with observations and the size of those pixels.[3] First, when observational coverage is low, inequalities are more likely to be based on census tracts having more residents in the majority population group with respect to UA area, typically non-Hispanic/Latino white residents (Table Appendix S2), a sampling bias that leads to population-weighted inequalities being underestimated.[3] Here, minimum daily coverage thresholds are identified by comparing relative XCO_{norm} and $XNO_{2,norm}$ inequalities at thresholds of 10%, 20%, and 40% to those at 60% coverage using the Wilcoxon rank sum test, a non-parametric two-sample t-test. Coverage requirements for daily mean annual and seasonal XCO and XNO_2 are typically 10%. Coverage thresholds are similar when based on relative CO VCD_{norm} and NO_2 $TVCD_{norm}$ inequalities, so we apply the XCO and XNO_2 thresholds for consistency.

Second, TROPOMI pixel sizes are variable: 40–240 km^2 for CO (median of 67 km^2) and 20–100 km^2 for NO_2 (median of 34 km^2) in the six UAs. We test the sensitivity of census tract-scale XCO_{norm} and $XNO_{2,norm}$ inequalities to pixel size, binning observations into pixel size terciles and comparing inequalities in larger pixel size bins to those in the lowest pixel size tercile (Wilcoxon rank sum test). We find annual mean daily XCO_{norm} and $XNO_{2,norm}$ inequalities are insensitive to pixel size in each UA (Table S5). Dressel et al.[3] reported NO_2 inequalities in New York City–Newark were biased low for pixels larger than 60 km^2 but insensitive to pixel size in Houston,[4] suggesting inequalities based on CO and NO_2 and/or $XNO_{2,norm}$ and NO_2 TVCDs are potentially differently sensitive to pixel size, although the analyses are not directly comparable because observations were subset into different pixel size bins.

Table Appendix S2. Mean daily relative inequalities in the frequency of days with XCO and XNO_2 observations for primarily Black, Latino, and Asian census tracts compared to primarily non-Hispanic/Latino white census tracts in Los Angeles–Riverside, Houston, Phoenix, Chicago, New York City–Newark, and Atlanta. Uncertainties are the 1σ standard error of the mean.

	Relative Inequalities (%)					
**Population Group**	**Los Angeles–Riverside**	**Houston**	**Phoenix**	**Chicago**	**New York City–Newark**	**Atlanta**
**Days with XCO and XNO_2**						
Black	–1 ± 1	–2 ± 1	0 ± 1	0 ± 1	–3 ± 1	0 ± 1
Latino	–1 ± 1	–3 ± 1	–1 ± 1	0 ± 1	–6 ± 1	–3 ± 1
Asian	1 ± 1	0 ± 1	0 ± 1	–3 ± 1	–1 ± 1	–2 ± 1

Table S3. Inequalities in XCO_{norm} over June 2018–February 2023 (excluding March 2020–February 2021) at 10%, 20%, 40%, and 60% coverage thresholds. Uncertainties are the 1σ standard error of the mean.

	10%	20%	40%	60%
**Population Group**		**Inequalities (%)**		
**Los Angeles–Riverside**				
Black	7 ± 1	7 ± 1	7 ± 1	7 ± 1
Latino	15 ± 1	15 ± 1	15 ± 1	15 ± 1
Asian	11 ± 1	11 ± 1	12 ± 1	12 ± 1
**Houston**				
Black	3 ± 2	5 ± 1	5 ± 1	6 ± 1
Latino	5 ± 2	7 ± 1	7 ± 1	8 ± 1
Asian	–5 ± 4	–2 ± 1	–2 ± 1	–2 ± 1
**Phoenix**				
Black	10 ± 1	9 ± 1	10 ± 1	10 ± 1
Latino	15 ± 1	15 ± 1	15 ± 1	15 ± 1
Asian	2 ± 1	2 ± 1	2 ± 1	2 ± 1
**Chicago**				
Black	10 ± 1	11 ± 1	12 ± 1	13 ± 1
Latino	7 ± 1	7 ± 1	8 ± 1	9 ± 1
Asian	1 ± 1	0 ± 1	0 ± 1	1 ± 1
**New York City–Newark**				
Black	11 ± 1	12 ± 1	11 ± 1	11 ± 1
Latino	8 ± 1	9 ± 1	9 ± 1	9 ± 1
Asian	8 ± 1	9 ± 1	9 ± 1	10 ± 1
**Atlanta**				
Black	1 ± 1	2 ± 1	2 ± 1	4 ± 1
Latino	3 ± 1	4 ± 1	4 ± 1	4 ± 1
Asian	4 ± 1	4 ± 1	4 ± 1	4 ± 1

Table S4. Inequalities in $XNO_{2,norm}$ over June 2018–February 2023 (excluding March 2020–February 2021) at 10%, 20%, 40%, and 60% coverage thresholds. Uncertainties are the 1σ standard error of the mean.

	**10%**	**20%**	**40%**	**60%**
**Population Group**	**Inequalities (%)**			
**Los Angeles–Riverside**				
Black	27 ± 1	27 ± 1	27 ± 1	26 ± 1
Latino	34 ± 1	35 ± 1	35 ± 1	35 ± 1
Asian	23 ± 1	23 ± 1	23 ± 1	24 ± 1
**Houston**				
Black	14 ± 1	14 ± 1	14 ± 1	13 ± 1
Latino	27 ± 1	29 ± 1	28 ± 1	29 ± 1
Asian	–1 ± 1	–1 ± 1	–1 ± 1	–2 ± 1
**Phoenix**				
Black	35 ± 1	36 ± 1	36 ± 1	36 ± 1
Latino	52 ± 1	53 ± 1	54 ± 1	54 ± 1
Asian	10 ± 1	10 ± 1	10 ± 1	10 ± 1
**Chicago**				
Black	23 ± 1	22 ± 1	23 ± 1	22 ± 1
Latino	21 ± 1	21 ± 1	22 ± 1	22 ± 1
Asian	8 ± 1	7 ± 1	6 ± 1	6 ± 1
**New York City–Newark**				
Black	28 ± 1	28 ± 1	28 ± 1	28 ± 1
Latino	26 ± 1	26 ± 1	25 ± 1	25 ± 1
Asian	26 ± 1	26 ± 1	26 ± 1	26 ± 1
**Atlanta**				
Black	32 ± 1	33 ± 1	35 ± 1	36 ± 1
Latino	13 ± 1	13 ± 1	13 ± 1	14 ± 1
Asian	9 ± 1	8 ± 1	8 ± 1	8 ± 1

Table S5. Mean census tract-scale inequalities in XCO_{norm} and $XNO_{2,norm}$ as a function of TROPOMI pixel area for primarily Black, Latino, and Asian compared to non-Hispanic/Latino white census tracts in Los Angeles–Riverside, Houston, Phoenix, Chicago, New York City–Newark, and Atlanta. Pixel areas are binned by terciles based on UA-specific size distributions, with inequalities calculated separately for the lowest third, lowest two-thirds, and all pixels. Low coverage days are removed.

	Mean XCO_{norm} Relative Inequalities (%)				Mean $XNO_{2,norm}$ Relative Inequalities (%)			
		Population Group				Population Group		
	Mean CO Pixel Areas (km^2)	Black	Latino	Asian	Mean NO_2 Pixel Areas (km^2)	Black	Latino	Asian
**Los Angeles–Riverside**								
	≤ first tercile	7 ± 1	14 ± 1	12 ± 1	≤ first tercile	26 ± 1	33 ± 1	22 ± 1
	≤ second tercile	7 ± 1	15 ± 1	11 ± 1	≤ second tercile	26 ± 1	34 ± 1	23 ± 1
	all pixels	7 ± 1	15 ± 1	11 ± 1	all pixels	27 ± 1	34 ± 1	23 ± 1
**Houston**								
	≤ first tercile	3 ± 1	6 ± 1	–3 ± 2	≤ first tercile	13 ± 1	26 ± 2	–1 ± 1
	≤ second tercile	5 ± 1	7 ± 1	–2 ± 1	≤ second tercile	14 ± 1	27 ± 1	–1 ± 1
	all pixels	5 ± 1	7 ± 1	–2 ± 1	all pixels	14 ± 1	27 ± 1	–1 ± 1
**Phoenix**								
	≤ first tercile	8 ± 1	13 ± 2	2 ± 1	≤ first tercile	33 ± 2	48 ± 2	10 ± 1
	≤ second tercile	10 ± 1	15 ± 1	2 ± 1	≤ second tercile	35 ± 1	52 ± 1	10 ± 1
	all pixels	10 ± 1	15 ± 1	2 ± 1	all pixels	35 ± 1	52 ± 1	10 ± 1
**Chicago**								
	≤ first tercile	8 ± 2	6 ± 2	0 ± 1	≤ first tercile	21 ± 2	21 ± 2	8 ± 2
	≤ second tercile	9 ± 2	7 ± 1	1 ± 1	≤ second tercile	21 ± 2	20 ± 1	8 ± 1
	all pixels	10 ± 1	7 ± 1	1 ± 1	all pixels	22 ± 1	20 ± 1	7 ± 1
**New York City–Newark**								
	≤ first tercile	11 ± 2	8 ± 1	10 ± 1	≤ first tercile	29 ± 1	25 ± 1	27 ± 1
	≤ second tercile	11 ± 1	9 ± 1	9 ± 1	≤ second tercile	28 ± 1	26 ± 1	26 ± 1
	all pixels	10 ± 1	8 ± 1	8 ± 1	all pixels	28 ± 1	26 ± 1	26 ± 1
**Atlanta**								
	≤ first tercile	3 ± 2	5 ± 1	6 ± 1	≤ first tercile	30 ± 2	14 ± 1	10 ± 1
	≤ second tercile	2 ± 1	3 ± 1	4 ± 1	≤ second tercile	32 ± 1	13 ± 1	9 ± 1
	all pixels	1 ± 1	3 ± 1	4 ± 1	all pixels	33 ± 1	13 ± 1	9 ± 1

Table S6. Percentage of days removed in Table 3, defined as days with one or more of the following conditions: low TROPOMI coverage in the UA; a $\Delta XNO_2/\Delta XCO$ Pearson correlation coefficient less than 0; statistically insignificant $\Delta XNO_2/\Delta XCO$ ($p \geq 0.05$); and/or no absolute XCO or XNO_2 inequalities.

	**Weekdays**	**Weekends**
Los Angeles–Riverside	12%	35%
Houston	68%	82%
Phoenix	32%	46%
Chicago	69%	67%
New York City–Newark	60%	69%
Atlanta	80%	89%

Appendix S3. Most routine CO instruments in Los Angeles–Riverside use non-dispersion cross-modulation infrared absorption (NDIR) with a reported detection limit of 0.5 ppm, which is higher than the free-troposphere CO background of ~70 ppb.[6] All other CO monitors included in this study use gas-filter correlation (GFC) with a lower detection limit, although data may be provided with a coarser resolution than possible analytically. Two locations in Los Angeles–Riverside operate both NDIR and GFC instruments, in which case we use the GFC measurements: for NDIR versus GFC, the mean slope is 1.1 (NDIR on *y*-axis) with $r = 0.88$.

NO_2* is mostly measured by chemiluminescence following catalytic decomposition of NO_2 to NO. This technique has a known positive interference, as a portion of organic nitrates, nitric acid, and ammonia also decompose across the catalyst at non-unity efficiency.[7-9] Because of this interference, which has a larger effect on accuracy than precision, we use the term NO_2*.[10]

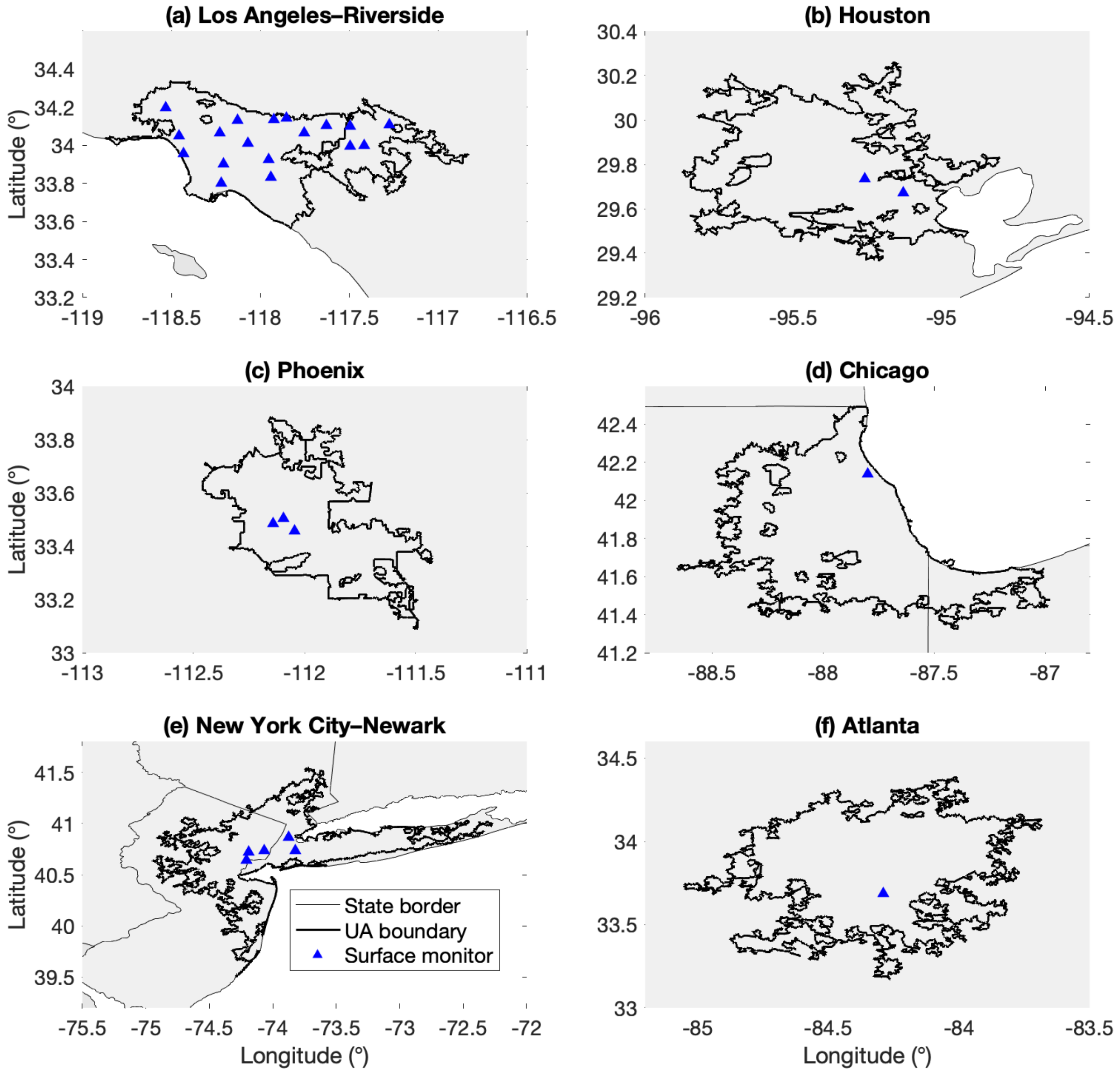

Figure S4. Co-located, non-roadway CO and NO_2* surface monitors with measurements in 2018–2023 in Los Angeles–Riverside (a), Houston (b), Phoenix (c), Chicago (d), New York City–Newark (e), and Atlanta (f). Thick black outlines are UA boundaries. Thin black lines are state borders.

Table S7. Locations of co-located non-roadway CO and NO_2* surface monitors in the UAs used to compare with TROPOMI $\Delta XNO_2/\Delta XCO$ in Table S18. Sites with non-dispersion cross-modulation infrared absorption (NDIR) instruments are indicated by a square (□). Sites with gas-filter correlation (GFC) instruments are indicated by a circle (○). When a site has both types, the GFC dataset is used in the analysis.

UA (Number of monitors)	Monitor Location	Temporal Coverage
**Los Angeles–Riverside (18)**		
□	–118.5328, 34.1993	2018–2023
□ ○	–118.2269, 34.0666	2018–2023
□	–118.2050, 33.9014	2018–2023
□	–118.1272, 34.1326	2018–2023
□	–118.0685, 34.0103	2018–2023
□	–117.9526, 33.9251	2018–2023
□	–117.9385, 33.8306	2018–2023
□	–117.8504, 34.1444	2018–2023
□	–117.7514, 34.0670	2018–2023
□	–117.6291, 34.1037	2018–2023
□	–117.4924, 33.9964	2018–2023
□	–117.4920, 34.1000	2018–2023
□ ○	–117.4160, 33.9996	2018–2023
○	–117.2741, 34.1067	2018–2023
□	–117.9239, 34.1365	2018–2022
□	–118.4564, 34.0511	2018–2021
□	–118.4305, 33.9551	2018–2021
□	–118.2200, 33.8025	2018–2019
**Houston (2)**		
○	–95.2576, 29.7337	2018–2023
○	–95.1285, 29.6700	2018–2023
**Phoenix (3)**		
○	–112.1426, 33.4838	2018–2023
○	–112.0958, 33.5038	2018–2023
○	–112.0466, 33.4580	2018–2023
**Chicago (1)**		
○	–87.7992, 42.1400	2018–2023
**New York City–Newark (5)**		
○	–74.2084, 40.6414	2018–2023
○	–74.0663, 40.7316	2018–2023
○	–73.8781, 40.8679	2018–2023
○	–73.8215, 40.7361	2018–2023
○	–74.1929, 40.7210	2018–2022
**Atlanta (1)**		
○	–84.2905, 33.6878	2018–2023

Table S8. Locations of non-roadway CO and NO_2* surface monitors within the CBSAs of Los Angeles–Riverside and Houston, in addition to those listed in Table S7. Co-location between CO and NO_2* is not required.

CBSA	Monitor Location	Measurements Used
**Los Angeles–Riverside**		
	–118.5284, 34.3834	NO_2*, CO
	–118.1305, 34.6697	NO_2*, CO
	–117.3714, 35.7742	NO_2*
	–117.3310, 33.6765	NO_2*, CO
	–117.3255, 34.5110	NO_2*, CO
	–117.0886, 33.4479	NO_2*
	–117.0248, 34.8940	NO_2*, CO
	–116.8584, 33.9209	NO_2*
	–116.8301, 33.9447	NO_2*
	–116.5410, 33.8528	NO_2*, CO
	–118.3630, 34.1820	NO_2*
	–118.1710, 33.7937	NO_2*
	–118.1786, 34.7254	NO_2*
	–117.6759, 33.6300	CO
**Houston**		
	–95.6740, 30.0395	NO_2*
	–95.4992, 29.6957	NO_2*
	–95.4892, 29.8342	NO_2*
	–95.4729, 29.0438	NO_2*
	–95.4251, 30.3503	NO_2*
	–95.3925, 29.5204	NO_2*
	–95.3261, 29.9010	NO_2*
	–95.2947, 29.6864	NO_2*
	–95.2206, 29.7680	NO_2*
	–95.1255, 29.8027	NO_2*
	–95.0794, 29.7589	NO_2*
	–95.0155, 29.5830	NO_2*
	–94.8613, 29.2545	NO_2*
	–95.3958, 29.7729	NO_2*

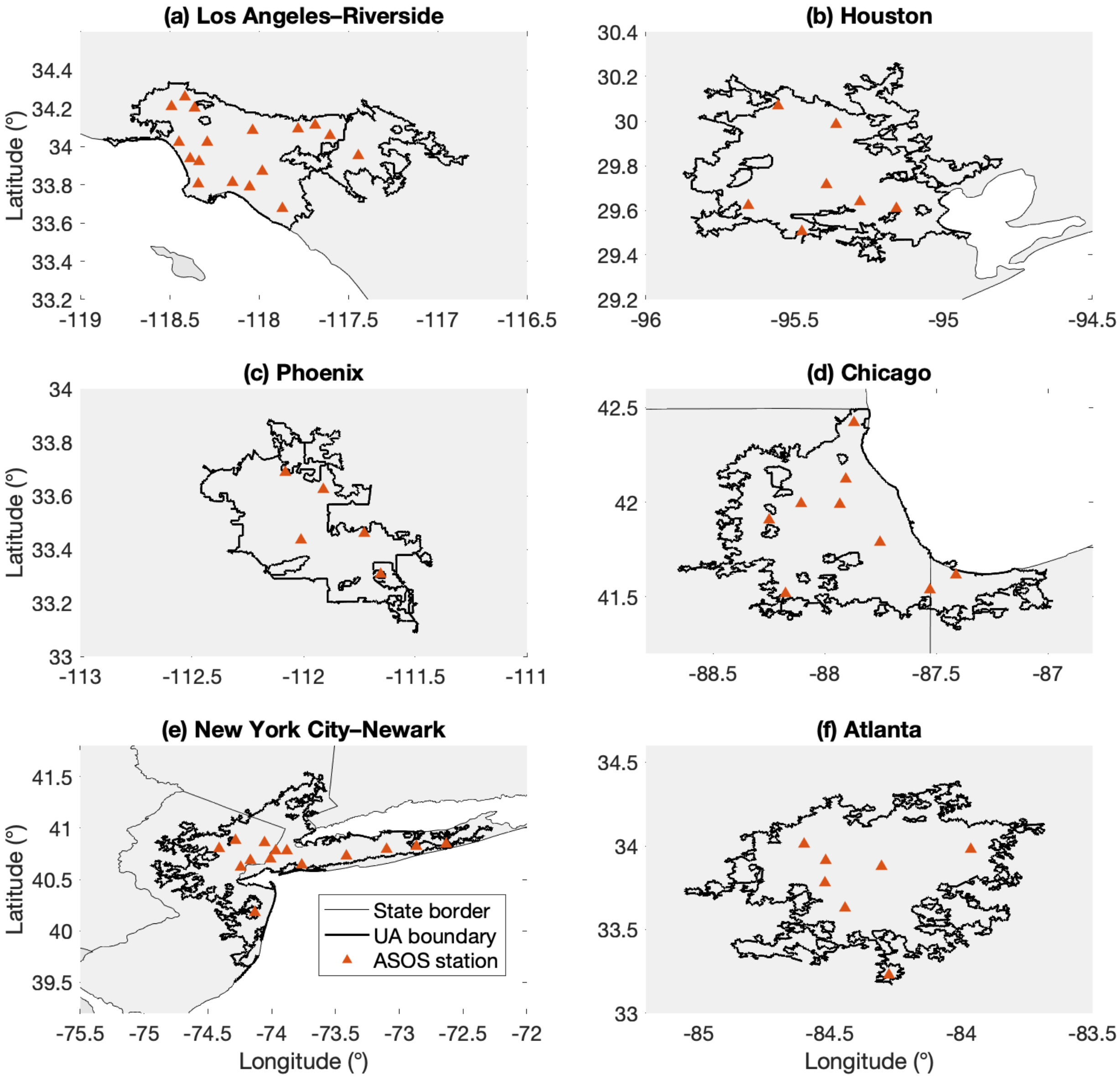

Figure S5. ASOS stations with wind measurements in 2018–2023 in Los Angeles–Riverside (a), Houston (b), Phoenix (c), Chicago (d), New York City–Newark (e), and Atlanta (f). Thick black outlines are UA boundaries. Thin black lines are state borders.

Table S9. Mean daily census tract-scale absolute inequalities in XCO, CO VCDs, XNO_2, and NO_2 TVCDs over June 2018–February 2023 (excluding March 2020–February 2021) for Black, Latino, and Asian compared to non-Hispanic/Latino white population groups in Los Angeles–Riverside, Houston, Phoenix, Chicago, New York City–Newark, and Atlanta. Uncertainties are the 1σ standard error of the mean.

	**XCO**	**CO VCDs**	**XNO_2**	**NO_2 TVCDs**
**Population Group**	**Inequalities (ppb)**			
**Los Angeles–Riverside**				
Black	0.5 ± 0.1	2.0 ± 0.2	0.08 ± 0.01	17 ± 1
Latino	1.3 ± 0.1	3.0 ± 0.1	0.10 ± 0.01	21 ± 1
Asian	0.9 ± 0.1	3.2 ± 0.1	0.07 ± 0.01	15 ± 1
**Houston**				
Black	0.3 ± 0.1	0.6 ± 0.1	0.02 ± 0.01	4 ± 1
Latino	0.4 ± 0.1	0.9 ± 0.1	0.03 ± 0.01	7 ± 1
Asian	–0.1 ± 0.1	–0.3 ± 0.1	0.00 ± 0.01	0 ± 1
**Phoenix**				
Black	0.5 ± 0.1	2.1 ± 0.1	0.05 ± 0.01	10 ± 1
Latino	0.8 ± 0.1	2.8 ± 0.1	0.07 ± 0.01	15 ± 1
Asian	0.1 ± 0.1	0.7 ± 0.1	0.01 ± 0.01	3 ± 1
**Chicago**				
Black	0.7 ± 0.1	2.0 ± 0.2	0.04 ± 0.01	9 ± 1
Latino	0.5 ± 0.1	1.3 ± 0.1	0.04 ± 0.01	8 ± 1
Asian	0.1 ± 0.1	0.1 ± 0.1	0.01 ± 0.01	3 ± 1
**New York City–Newark**				
Black	0.8 ± 0.1	2.2 ± 0.1	0.08 ± 0.01	18 ± 1
Latino	0.7 ± 0.1	1.7 ± 0.1	0.08 ± 0.01	16 ± 1
Asian	0.6 ± 0.1	1.8 ± 0.1	0.07 ± 0.01	16 ± 1
**Atlanta**				
Black	0.0 ± 0.1	0.6 ± 0.1	0.03 ± 0.01	6 ± 1
Latino	0.2 ± 0.1	0.5 ± 0.1	0.01 ± 0.01	2 ± 1
Asian	0.3 ± 0.1	0.5 ± 0.1	0.01 ± 0.01	2 ± 1

Table S10. Mean daily relative inequalities in the frequency of days with XCO and XNO_2 above the median and seventieth percentiles of daily observations for Black, Latino, and Asian population groups compared to the non-Hispanic/Latino white group in Los Angeles–Riverside, Houston, Phoenix, Chicago, New York City–Newark, and Atlanta. Uncertainties are the 1σ standard error of the mean.

	Inequality (%)					
**Population Group**	**Los Angeles–Riverside**	**Houston**	**Phoenix**	**Chicago**	**New York City–Newark**	**Atlanta**
**Days above Median XCO**						
Black	7 ± 1	3 ± 1	12 ± 2	17 ± 1	12 ± 1	4 ± 1
Latino	21 ± 1	5 ± 1	20 ± 2	14 ± 1	5 ± 1	2 ± 1
Asian	16 ± 1	–5 ± 1	2 ± 2	–2 ± 1	12 ± 1	3 ± 1
**Days above the 70th-Percentile XCO**						
Black	4 ± 2	1 ± 1	12 ± 2	21 ± 1	8 ± 1	4 ± 1
Latino	23 ± 2	5 ± 1	24 ± 2	15 ± 1	2 ± 1	2 ± 1
Asian	21 ± 2	–10 ± 1	1 ± 3	–1 ± 1	8 ± 1	2 ± 1
**Days above Median XNO_2**						
Black	50 ± 2	15 ± 3	49 ± 4	43 ± 2	43 ± 2	45 ± 3
Latino	55 ± 2	31 ± 2	63 ± 3	44 ± 3	35 ± 2	18 ± 3
Asian	38 ± 2	–7 ± 3	17 ± 4	5 ± 3	47 ± 2	8 ± 3
**Days above the 70th-Percentile XNO_2**						
Black	62 ± 3	23 ± 4	76 ± 6	40 ± 3	50 ± 2	69 ± 4
Latino	76 ± 3	45 ± 3	95 ± 5	48 ± 3	37 ± 3	22 ± 5
Asian	60 ± 3	–16 ± 4	27 ± 7	14 ± 3	51 ± 2	9 ± 5

Equation S4. The hydrostatic equation calculates atmospheric pressure (p) at a given elevation (h). p_0 is the atmospheric pressure at sea level (101.325 kPa), g is the gravitational acceleration constant (9.81 m s^{-1}), M is the molar mass of dry air (0.02897 kg mol^{-1}), h_0 is the reference height (in this case, sea level), R is the ideal gas constant (8.314 J K^{-1} mol^{-1}), and T is the air temperature at elevation h, assumed to be 298 K.

(Eq. S4) $$p = p_0 \mathrm{e}^{-\frac{gM(h-h_0)}{\mathrm{R}T}}$$

Table S11. Inequalities in XCO_{norm} and $XNO_{2,norm}$ (%) and absolute inequalities in XCO and XNO_2 (ppb) on calm (below median) and windy (above median) days based on mean daily (12–3 pm LT) UA wind speeds. Mean daily temperatures (T) and wind speeds (WS) are shown under XCO_{norm}.

	XCO_{norm}		$XNO_{2,norm}$		XCO		XNO_2	
**Population Group**	**Calm days**	**Windy days**	**Calm days**	**Windy days**	**Calm days**	**Windy days**	**Calm days**	**Windy days**
	**Relative Inequalities (%)**				**Absolute Inequalities (ppb)**			
**Los Angeles–Riverside**	T: 21°C WS: 3.0 m/s	T: 25°C WS: 4.5 m/s						
Black	10 ± 1	4 ± 1	28 ± 1	25 ± 1	0.9 ± 0.1	0.2 ± 0.1	0.11 ± 0.01	0.06 ± 0.01
Latino	14 ± 1	16 ± 1	30 ± 1	38 ± 1	1.3 ± 0.1	1.3 ± 0.1	0.11 ± 0.01	0.09 ± 0.01
Asian	10 ± 1	12 ± 1	18 ± 1	26 ± 1	0.9 ± 0.1	0.9 ± 0.1	0.07 ± 0.01	0.06 ± 0.01
**Houston**	T: 27°C WS: 2.6 m/s	T: 24°C WS: 5.4 m/s						
Black	6 ± 1	–1 ± 5	17 ± 1	10 ± 1	0.4 ± 0.1	0.2 ± 0.1	0.02 ± 0.01	0.01 ± 0.01
Latino	9 ± 1	1 ± 4	32 ± 1	21 ± 1	0.5 ± 0.1	0.2 ± 0.1	0.04 ± 0.01	0.02 ± 0.01
Asian	0 ± 1	–12 ± 9	2 ± 1	–6 ± 1	–0.1 ± 0.1	–0.2 ± 0.1	0.00 ± 0.01	0.00 ± 0.01
**Phoenix**	T: 26°C WS: 2.0 m/s	T: 29°C WS: 3.9 m/s						
Black	16 ± 1	3 ± 1	47 ± 1	23 ± 1	0.9 ± 0.1	0.2 ± 0.1	0.07 ± 0.01	0.02 ± 0.01
Latino	24 ± 1	6 ± 1	68 ± 1	36 ± 2	1.4 ± 0.1	0.3 ± 0.1	0.11 ± 0.01	0.03 ± 0.01
Asian	5 ± 1	0 ± 1	14 ± 1	5 ± 1	0.3 ± 0.1	0.0 ± 0.1	0.02 ± 0.01	0.01 ± 0.01
**Chicago**	T: 16°C WS: 2.8 m/s	T: 13°C WS: 5.7 m/s						
Black	13 ± 2	7 ± 2	23 ± 2	23 ± 2	1.1 ± 0.2	0.4 ± 0.1	0.05 ± 0.01	0.03 ± 0.01
Latino	8 ± 1	6 ± 1	22 ± 1	20 ± 2	0.6 ± 0.1	0.4 ± 0.1	0.05 ± 0.01	0.02 ± 0.01
Asian	1 ± 1	0 ± 1	6 ± 1	11 ± 2	0.1 ± 0.1	0.0 ± 0.1	0.02 ± 0.01	0.01 ± 0.01
**New York City–Newark**	T: 17°C WS: 3.0 m/s	T: 15°C WS: 5.9 m/s						
Black	14 ± 1	8 ± 1	30 ± 1	27 ± 1	1.1 ± 0.1	0.6 ± 0.1	0.11 ± 0.01	0.05 ± 0.01
Latino	12 ± 1	5 ± 1	28 ± 1	24 ± 1	1.0 ± 0.1	0.4 ± 0.1	0.11 ± 0.01	0.05 ± 0.01
Asian	11 ± 1	6 ± 1	28 ± 1	24 ± 1	0.9 ± 0.1	0.4 ± 0.1	0.10 ± 0.01	0.05 ± 0.01
**Atlanta**	T: 23°C WS: 2.0 m/s	T: 19°C WS: 4.3 m/s						
Black	0 ± 1	2 ± 1	35 ± 1	30 ± 2	0.0 ± 0.1	0.1 ± 0.1	0.03 ± 0.01	0.02 ± 0.01
Latino	3 ± 1	4 ± 1	14 ± 1	12 ± 1	0.2 ± 0.1	0.2 ± 0.1	0.01 ± 0.01	0.01 ± 0.01
Asian	4 ± 1	4 ± 1	8 ± 1	10 ± 1	0.3 ± 0.1	0.2 ± 0.1	0.01 ± 0.01	0.01 ± 0.01

Table S12. Spearman's rank correlation coefficients between mean daily absolute inequalities in XCO and XNO_2 and mean daily (12–3 pm LT) UA wind speeds and temperatures. Only statistically significant coefficients ($p < 0.05$) are shown.

	**Absolute XCO Inequalities (ppb)**		**Absolute XNO_2 Inequalities (ppb)**	
	**Wind speed**	**Temperature**	**Wind speed**	**Temperature**
**Population Group**	**Spearman's rank correlation coefficient (ρ)**			
**Los Angeles–Riverside**				
Black	–0.16	–0.23	–0.35	–0.13
Latino			–0.16	
Asian	–0.03		–0.04	
**Houston**				
Black	–0.13	–0.08	–0.38	
Latino	–0.19		–0.52	
Asian		–0.22	–0.16	0.12
**Phoenix**				
Black	–0.34	–0.31	–0.64	–0.21
Latino	–0.36	–0.29	–0.65	–0.17
Asian	–0.23	–0.23	–0.54	–0.23
**Chicago**				
Black	–0.16		–0.22	–0.15
Latino	–0.13		–0.38	
Asian	–0.03			
**New York City–Newark**				
Black	–0.22		–0.40	–0.21
Latino	–0.25		–0.38	
Asian	–0.27		–0.46	–0.10
**Atlanta**				
Black		–0.10	–0.14	–0.22
Latino	–0.05		–0.15	–0.15
Asian	–0.07		–0.08	

Table S13. Inequalities in XCO_{norm} and $XNO_{2,norm}$ (%) and absolute inequalities in XCO and XNO_2 (ppb) on cool (below median) and hot (above median) days based on mean daily (12–3 pm LT) UA temperatures. Mean daily temperatures (T) and wind speeds (WS) are shown under XCO_{norm}.

	**XCO_{norm}**		**$XNO_{2,norm}$**		**XCO**		**XNO_2**	
	**Cool days**	**Hot days**	**Cool days**	**Hot days**	**Cool days**	**Hot days**	**Cool days**	**Hot days**
**Population Group**	**Relative Inequalities (%)**				**Absolute Inequalities (ppb)**			
**Los Angeles–Riverside**	T: 18°C WS: 3.5 m/s	T: 28°C WS: 4.0 m/s						
Black	12 ± 1	3 ± 1	27 ± 1	27 ± 1	1.0 ± 0.1	0.2 ± 0.1	0.09 ± 0.01	0.07 ± 0.01
Latino	15 ± 1	15 ± 1	32 ± 1	37 ± 1	1.3 ± 0.1	1.3 ± 0.1	0.10 ± 0.01	0.09 ± 0.01
Asian	12 ± 1	10 ± 1	20 ± 1	24 ± 1	1.0 ± 0.1	0.9 ± 0.1	0.07 ± 0.01	0.07 ± 0.01
**Houston**	T: 19°C WS: 4.2 m/s	T: 31°C WS: 3.6 m/s						
Black	8 ± 1	0 ± 4	10 ± 1	17 ± 1	0.4 ± 0.1	0.2 ± 0.1	0.02 ± 0.01	0.01 ± 0.01
Latino	7 ± 1	4 ± 3	22 ± 1	31 ± 1	0.3 ± 0.1	0.4 ± 0.1	0.04 ± 0.01	0.03 ± 0.01
Asian	4 ± 1	–11 ± 6	–4 ± 1	1 ± 1	0.1 ± 0.1	–0.3 ± 0.1	0.00 ± 0.01	0.00 ± 0.01
**Phoenix**	T: 20°C WS: 2.8 m/s	T: 35°C WS: 3.0 m/s						
Black	17 ± 1	3 ± 1	38 ± 1	33 ± 1	0.9 ± 0.1	0.2 ± 0.1	0.06 ± 0.01	0.03 ± 0.01
Latino	23 ± 1	7 ± 1	54 ± 2	51 ± 1	1.3 ± 0.1	0.4 ± 0.1	0.09 ± 0.01	0.05 ± 0.01
Asian	5 ± 1	0 ± 1	12 ± 1	8 ± 1	0.3 ± 0.1	0.0 ± 0.1	0.02 ± 0.01	0.01 ± 0.01
**Chicago**	T: 4°C WS: 4.6 m/s	T: 24°C WS: 4.0 m/s						
Black	16 ± 2	7 ± 2	28 ± 3	20 ± 1	1.0 ± 0.1	0.6 ± 0.1	0.06 ± 0.01	0.03 ± 0.01
Latino	8 ± 1	7 ± 1	19 ± 2	22 ± 1	0.5 ± 0.1	0.5 ± 0.1	0.05 ± 0.01	0.04 ± 0.01
Asian	1 ± 1	1 ± 1	9 ± 2	7 ± 1	0.1 ± 0.1	0.1 ± 0.1	0.02 ± 0.01	0.01 ± 0.01
**New York City–Newark**	T: 8°C WS: 4.8 m/s	T: 25°C WS: 4.1 m/s						
Black	12 ± 1	10 ± 1	29 ± 1	27 ± 1	0.9 ± 0.1	0.8 ± 0.1	0.10 ± 0.01	0.07 ± 0.01
Latino	7 ± 1	9 ± 1	23 ± 1	28 ± 1	0.6 ± 0.1	0.7 ± 0.1	0.08 ± 0.01	0.07 ± 0.01
Asian	7 ± 1	9 ± 1	24 ± 1	27 ± 1	0.6 ± 0.1	0.7 ± 0.1	0.08 ± 0.01	0.07 ± 0.01
**Atlanta**	T: 14°C WS: 3.5 m/s	T: 28°C WS: 2.8 m/s						
Black	6 ± 1	–2 ± 1	34 ± 2	32 ± 1	0.3 ± 0.1	–0.1 ± 0.1	0.04 ± 0.01	0.02 ± 0.01
Latino	5 ± 1	2 ± 1	13 ± 1	14 ± 1	0.3 ± 0.1	0.2 ± 0.1	0.01 ± 0.01	0.01 ± 0.01
Asian	4 ± 1	4 ± 1	7 ± 1	10 ± 1	0.3 ± 0.1	0.3 ± 0.1	0.01 ± 0.01	0.01 ± 0.01

Table S14. Inequalities in XCO_{norm} and $XNO_{2,norm}$ (%) and absolute inequalities in XCO and XNO_2 (ppb) in summer (June–August) and winter (December–February) for Black, Latino, and Asian population groups compared to non-Hispanic/Latino white residents in Los Angeles–Riverside, Houston, Phoenix, Chicago, New York City–Newark, and Atlanta. Uncertainties are the 1σ standard error of the mean. Mean daily (12–3 pm LT) UA wind speeds and median wind directions with their 1σ standard deviations are also shown.

			Summer			
**Population Group**	**XCO_{norm} Inequalities (%)**	**$XNO_{2,norm}$ Inequalities (%)**	**Absolute XCO Inequalities (ppb)**	**Absolute XNO_2 Inequalities (ppb)**	**Wind speed ($m\ s^{-1}$)**	**Wind direction (degree)**
**Los Angeles–Riverside**					4.3 ± 0.5	235 ± 13
Black	2 ± 1	30 ± 1	0.1 ± 0.1	0.06 ± 0.01		
Latino	18 ± 1	43 ± 1	1.4 ± 0.1	0.09 ± 0.01		
Asian	9 ± 1	26 ± 1	0.7 ± 0.1	0.05 ± 0.01		
**Houston**					3.2 ± 1.3	160 ± 68
Black	–3 ± 7	18 ± 1	0.2 ± 0.1	0.01 ± 0.01		
Latino	3 ± 5	33 ± 1	0.5 ± 0.1	0.02 ± 0.01		
Asian	–18 ± 11	3 ± 1	–0.4 ± 0.1	0.00 ± 0.01		
**Phoenix**					3.1 ± 0.9	223 ± 51
Black	–1 ± 1	32 ± 1	0.0 ± 0.1	0.03 ± 0.01		
Latino	3 ± 2	49 ± 2	0.2 ± 0.1	0.05 ± 0.01		
Asian	–2 ± 1	8 ± 1	–0.1 ± 0.1	0.01 ± 0.01		
**Chicago**					3.8 ± 1.4	173 ± 93
Black	7 ± 2	23 ± 2	0.6 ± 0.2	0.03 ± 0.01		
Latino	6 ± 1	25 ± 1	0.4 ± 0.1	0.04 ± 0.01		
Asian	–2 ± 1	4 ± 1	–0.2 ± 0.1	0.01 ± 0.01		
**New York City–Newark**					4.0 ± 1.2	200 ± 77
Black	9 ± 1	27 ± 1	0.8 ± 0.1	0.06 ± 0.01		
Latino	8 ± 1	28 ± 1	0.6 ± 0.1	0.06 ± 0.01		
Asian	7 ± 1	27 ± 1	0.6 ± 0.1	0.06 ± 0.01		
**Atlanta**					2.6 ± 1.0	200 ± 92
Black	–1 ± 2	35 ± 2	–0.1 ± 0.2	0.02 ± 0.01		
Latino	1 ± 1	14 ± 1	0.2 ± 0.1	0.01 ± 0.01		
Asian	3 ± 1	10 ± 1	0.3 ± 0.1	0.01 ± 0.01		

Winter						
**Population Group**	**XCO_{norm} Inequalities (%)**	**$XNO_{2,norm}$ Inequalities (%)**	**Absolute XCO Inequalities (ppb)**	**Absolute XNO_2 Inequalities (ppb)**	**Wind speed ($m\ s^{-1}$)**	**Wind direction (degree)**
**Los Angeles–Riverside**					3.1 ± 1.2	220 ± 66
Black	13 ± 2	25 ± 2	1.3 ± 0.1	0.11 ± 0.01		
Latino	12 ± 1	24 ± 1	1.1 ± 0.1	0.10 ± 0.01		
Asian	12 ± 1	18 ± 1	1.1 ± 0.1	0.07 ± 0.01		
**Houston**					4.2 ± 1.9	180 ± 108
Black	10 ± 2	9 ± 1	0.5 ± 0.1	0.02 ± 0.01		
Latino	10 ± 2	21 ± 1	0.5 ± 0.1	0.04 ± 0.01		
Asian	4 ± 2	–7 ± 2	0.2 ± 0.1	0.00 ± 0.01		
**Phoenix**					2.5 ± 1.4	160 ± 90
Black	19 ± 2	40 ± 2	1.1 ± 0.1	0.08 ± 0.01		
Latino	28 ± 2	57 ± 3	1.6 ± 0.1	0.11 ± 0.01		
Asian	7 ± 1	13 ± 1	0.4 ± 0.1	0.03 ± 0.01		
**Chicago**					4.5 ± 2.0	230 ± 94
Black	11 ± 3	28 ± 3	0.8 ± 0.2	0.07 ± 0.01		
Latino	8 ± 2	21 ± 2	0.6 ± 0.1	0.06 ± 0.01		
Asian	1 ± 2	12 ± 2	0.1 ± 0.1	0.02 ± 0.01		
**New York City–Newark**					4.5 ± 2.2	240 ± 101
Black	14 ± 2	29 ± 2	1.0 ± 0.1	0.10 ± 0.01		
Latino	9 ± 2	22 ± 2	0.8 ± 0.1	0.08 ± 0.01		
Asian	9 ± 1	24 ± 1	0.7 ± 0.1	0.08 ± 0.01		
**Atlanta**					3.5 ± 1.6	220 ± 98
Black	7 ± 2	30 ± 3	0.3 ± 0.1	0.04 ± 0.01		
Latino	5 ± 2	13 ± 1	0.2 ± 0.1	0.02 ± 0.01		
Asian	4 ± 2	8 ± 1	0.2 ± 0.1	0.01 ± 0.01		

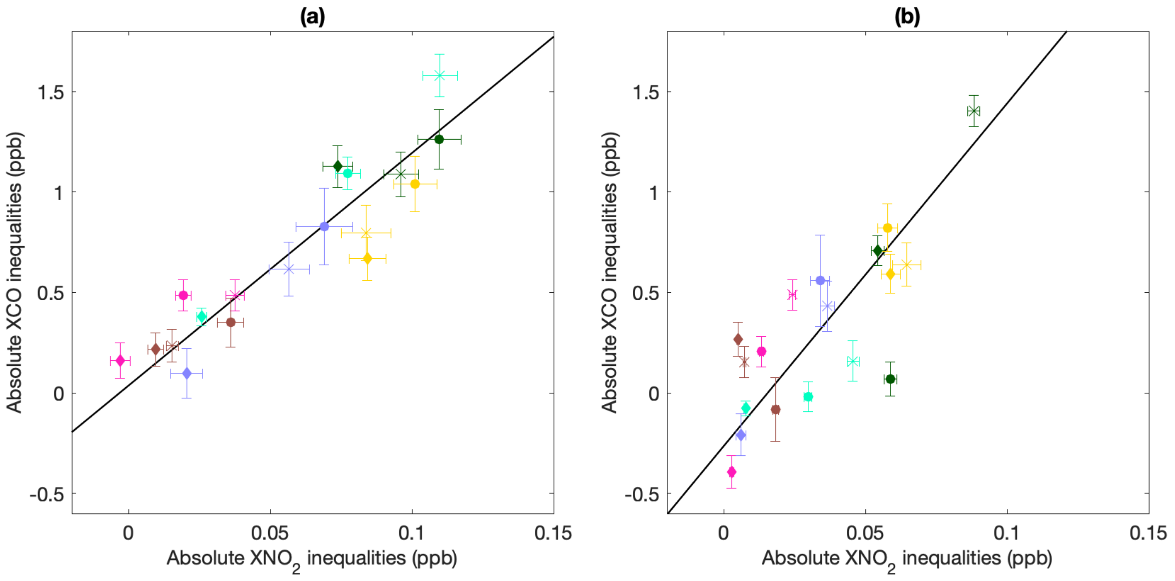

Figure S6. Mean daily census tract-scale absolute XCO and XNO_2 inequalities (ppb) in winter (a) and summer (b) months for Black (○), Latino (×), and Asian (◇) residents in Los Angeles–Riverside (dark green), Houston (magenta), Phoenix (cyan), Chicago (periwinkle), New York City–Newark (yellow), and Atlanta (brown). The reduced major-axis regression line (black) is $y = 11.6x + 0.04$ and $r = 0.92$ (winter) and $y = 17.1x - 0.27$ and $r = 0.79$ (summer). Error bars are uncertainties based on standard errors of the mean.

Table S15. Sector-wise NEI20 E_{NO2}/E_{CO} (molar basis), NO_2 and CO emissions (mass basis), and their percentages at the UA level in Los Angeles–Riverside, Houston, Phoenix, Chicago, New York City–Newark, and Atlanta.

	Total	Mobile Source	On-road Gas	On-road Diesel	Off-road Gas and Diesel	Stationary Source	Oil and Gas	Electricity Generation	Chemical Plant
**UA-wide NEI20 E_{NO2}/E_{CO}**									
Los Angeles–Riverside	0.084	0.072	0.041	2.266	0.028	0.275	0.521	0.115	0.305
Houston	0.083	0.058	0.020	0.739	0.023	0.455	0.635	0.430	0.772
Phoenix	0.063	0.047	0.028	0.746	0.043	0.479	–	2.728	–
Chicago	0.063	0.048	0.021	0.921	0.022	0.091	1.175	0.900	0.469
New York City–Newark	0.060	0.046	0.022	0.875	0.023	0.574	1.265	0.691	0.716
Atlanta	0.056	0.046	0.027	0.793	0.019	0.590	3.998	6.254	–
**UA-wide NO_2 emissions (unit: metric Ton)**									
Los Angeles–Riverside	36512	29722	6319	9990	6444	6790	2404	428	18
Houston	30863	20169	3206	6627	3914	10694	3177	1015	3791
Phoenix	10523	7429	2928	1982	2179	3094	0	1896	0
Chicago	41706	20644	4013	8006	4891	21062	1953	1181	448
New York City–Newark	53792	40325	6934	13295	12102	13467	635	5252	23
Atlanta	27072	21971	7151	10079	3632	5101	1576	336	0
**UA-wide CO emissions (unit: metric Ton)**									
Los Angeles–Riverside	265433	250417	93896	2684	140122	15015	2809	2269	35
Houston	225987	211685	97701	5462	103003	14302	3047	1435	2991
Phoenix	101139	97210	63454	1618	31182	3930	0	423	0
Chicago	401744	260118	114316	5293	136145	141626	1012	798	581
New York City–Newark	544673	530382	188089	9254	324334	14291	305	4624	19
Atlanta	294590	289325	159822	7732	119211	5265	240	33	0
**UA-wide NO_2 emissions (%)**									
Los Angeles–Riverside	100	81	17	27	18	19	7	1	0
Houston	100	65	10	21	13	35	10	3	12
Phoenix	100	71	28	19	21	29	0	18	0
Chicago	100	49	10	19	12	51	5	3	1
New York City–Newark	100	75	13	25	22	25	1	10	0
Atlanta	100	81	26	37	13	19	6	1	0
**UA-wide CO emissions (%)**									
Los Angeles–Riverside	100	94	35	1	53	6	1	1	0
Houston	100	94	43	2	46	6	1	1	1
Phoenix	100	96	63	2	31	4	0	0	0
Chicago	100	65	28	1	34	35	0	0	0
New York City–Newark	100	97	35	2	60	3	0	1	0
Atlanta	100	98	54	3	40	2	0	0	0

Equation S5. NEI20 NO_2/CO emission ratio on the molar basis (E_{NO2}/E_{CO}). M_{CO} is the molar mass of CO, M_{NO2} is the molar mass of NO_2, $E_{NOx,mass}$ is the NO_x emission reported on the mass basis, and $E_{CO,mass}$ is the CO emission reported on the mass basis. $E_{NOx,mass}$ is divided by a factor of 1.32 to convert to an effective NO_2 emission rate.

(Eq. S5)
$$\frac{E_{NO2}}{E_{CO}} = \left(\frac{M_{CO}}{M_{NO2}}\right)\frac{E_{NOx,mass}}{1.32E_{CO,mass}}$$

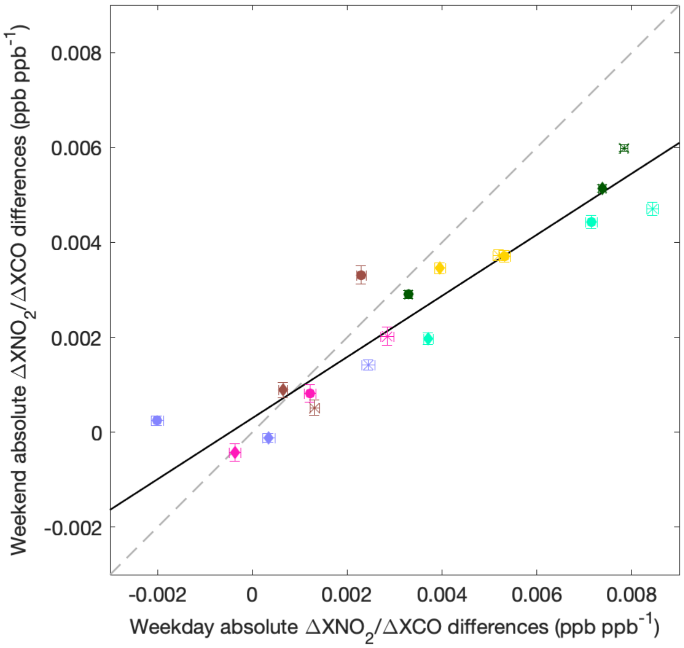

Figure S7. Differences in $\Delta XNO_2/\Delta XCO$ on weekends and weekdays for Black (○), Latino (×), and Asian (◇) residents in Los Angeles–Riverside (dark green), Houston (magenta), Phoenix (cyan), Chicago (periwinkle), New York City–Newark (yellow), and Atlanta (brown). The reduced major-axis regression line (black) is $y = 0.64x + 0.00$ and $r = 0.94$. The gray line is 1:1. Error bars are uncertainties propagated as derived from $1\sigma/N^{1/2}$ and often not large enough to be seen behind the data markers.

Table S16. Mean daily census tract-scale NO_2 and CO averaging kernels and percent differences in averaging kernels for Black, Latino, Asian, and non-Hispanic/Latino white census tracts in Los Angeles–Riverside, Houston, Phoenix, Chicago, New York City–Newark, and Atlanta in summer (June–August). Uncertainties are the 1σ standard error of the mean.

	NO_2 Averaging Kernels (unitless)		Percent Difference Relative to Non-Hispanic/Latino Whites (%)	
**Population Group**	**Lowest Layer**	**Mean of First Five Layers**	**Lowest Layer**	**Mean of First Five Layers**
**Los Angeles–Riverside**				
Black	0.57	0.79	9 ± 1	2 ± 1
Latino	0.55	0.78	6 ± 1	1 ± 1
Asian	0.50	0.78	2 ± 1	1 ± 1
Non-Hispanic White	0.48	0.77	–	–
**Houston**				
Black	0.44	0.59	2 ± 1	2 ± 1
Latino	0.44	0.59	1 ± 1	1 ± 1
Asian	0.44	0.59	2 ± 1	1 ± 1
Non-Hispanic White	0.43	0.58	–	–
**Phoenix**				
Black	0.60	0.72	6 ± 1	3 ± 1
Latino	0.61	0.73	6 ± 1	4 ± 1
Asian	0.57	0.70	2 ± 1	1 ± 1
Non-Hispanic White	0.55	0.69	–	–
**Chicago**				
Black	0.28	0.70	3 ± 1	4 ± 1
Latino	0.28	0.68	2 ± 1	2 ± 1
Asian	0.26	0.67	1 ± 1	1 ± 1
Non-Hispanic White	0.25	0.66	–	–
**New York City–Newark**				
Black	0.45	0.69	5 ± 1	4 ± 1
Latino	0.44	0.67	3 ± 1	2 ± 1
Asian	0.43	0.67	3 ± 1	2 ± 1
Non-Hispanic White	0.40	0.65	–	–
**Atlanta**				
Black	0.21	0.51	–3 ± 1	0 ± 1
Latino	0.24	0.51	0 ± 1	0 ± 1
Asian	0.26	0.50	2 ± 1	0 ± 1
Non-Hispanic White	0.24	0.51	–	–
	**CO Averaging Kernels (unitless)**		**Percent Difference Relative to Non-Hispanic/Latino Whites (%)**	
**Population Group**	**Lowest Layer**	**Mean of First Two Layers**	**Lowest Layer**	**Mean of First Two Layers**
**Los Angeles–Riverside**				
Black	0.82	0.87	1 ± 1	1 ± 1
Latino	0.82	0.87	1 ± 1	1 ± 1
Asian	0.82	0.87	1 ± 1	1 ± 1
Non-Hispanic White	0.81	0.87	–	–
**Houston**				
Black	0.42	0.57	0 ± 1	0 ± 1
Latino	0.43	0.58	1 ± 1	1 ± 1
Asian	0.42	0.57	1 ± 1	0 ± 1
Non-Hispanic White	0.42	0.57	–	–
**Phoenix**				
Black	0.79	0.84	0 ± 1	0 ± 1
Latino	0.79	0.84	0 ± 1	0 ± 1
Asian	0.79	0.85	0 ± 1	0 ± 1
Non-Hispanic White	0.79	0.85	–	–
**Chicago**				
Black	0.56	0.69	2 ± 1	1 ± 1
Latino	0.57	0.70	3 ± 1	2 ± 1
Asian	0.54	0.67	–1 ± 1	–1 ± 1
Non-Hispanic White	0.55	0.68	–	–
**New York City–Newark**				
Black	0.53	0.66	4 ± 1	2 ± 1
Latino	0.50	0.64	1 ± 1	1 ± 1
Asian	0.51	0.64	2 ± 1	1 ± 1
Non-Hispanic White	0.49	0.63	–	
**Atlanta**				
Black	0.37	0.53	0 ± 1	0 ± 1
Latino	0.37	0.53	0 ± 1	0 ± 1
Asian	0.37	0.53	0 ± 1	0 ± 1
Non-Hispanic White	0.37	0.53	–	–

Table S17. Correlations between mean daily (12–3 pm LT) surface NO_2* and CO and TROPOMI XNO_2 and XCO as a function of observation separation distance in the CBSAs of Los Angeles–Riverside and Houston. Correlation coefficients (r) are the mean values between TROPOMI and each monitor. Only monitors with statistically significant correlations ($p < 0.05$) are used to calculate the means, and the number of monitors used is shown in brackets for each case. No AK: Original TROPOMI observations are used without averaging kernels. Level 1: TROPOMI observations are divided by the bottom layer of the averaging kernels. With AK: TROPOMI observations are divided by the average of all averaging kernel layers within the ABL (NO_2: lowest five layers; CO: lowest two layers).

**Los Angeles–Riverside (total monitors in CBSA: NO_2 = 31, CO = 25)**			
Mean r between TROPOMI XNO_2 and Surface NO_2* (Number of Monitors)			
Radius	No AK	Level 1	With AK (Level 1–5)
1 km	0.66 (25)	0.61 (22)	0.66 (23)
2 km	0.60 (26)	0.52 (26)	0.56 (27)
5 km	0.58 (28)	0.51 (28)	0.57 (28)
10 km	0.59 (28)	0.52 (28)	0.57 (28)
Mean r between TROPOMI XCO and Surface CO (Number of Monitors)			
Radius	No AK	Level 1	With AK (Level 1–2)
1 km	0.52 (9)	0.35 (5)	0.55 (7)
2 km	0.27 (15)	0.18 (6)	0.21 (12)
5 km	0.25 (18)	0.16 (7)	0.17 (12)
10 km	0.26 (17)	0.15 (9)	0.16 (13)
**Houston (total monitors in CBSA: NO_2 = 16)**			
Mean r between TROPOMI XNO_2 and Surface NO_2* (Number of Monitors)			
Radius	No AK	Level 1	With AK (Level 1–5)
1 km	0.74 (11)	0.81 (8)	0.79 (9)
2 km	0.60 (15)	0.52 (16)	0.53 (16)
5 km	0.56 (15)	0.50 (16)	0.50 (16)
10 km	0.56 (16)	0.52 (16)	0.52 (16)

Table S18. TROPOMI $\Delta XNO_2/\Delta XCO$ and surface NO_2*/CO by city and monitor. TROPOMI $\Delta XNO_2/\Delta XCO$ are the mean daily slopes of XNO_2 against XCO within 5 km of each surface monitor minus their daily 20th percentiles using reduced major-axis regression. Surface NO_2*/CO are the seasonal slopes of mean daily (12–3 pm LT) NO_2* against CO using reduced major-axis regression. Days with low TROPOMI coverage are removed. Uncertainties are derived from mean daily standard errors of slopes and generally less than 1%. The mean of each city is shown in bold. Surface monitors with insignificant ($p \geq 0.05$) and/or non-positive NO_2*/CO correlations ($r \leq 0$) are marked with a # and not used to calculate the mean. The two monitors in Chicago and Atlanta are not shown due to insufficient TROPOMI observations.

	**Summer**	
**Surface Monitor**	**TROPOMI $\Delta XNO_2/\Delta XCO$**	**Surface NO_2*/CO**
**Los Angeles–Riverside (18 sites)**	**0.0425**	**0.0533**
–118.5328, 34.1993	0.0188	0.0193
–118.2269, 34.0666	0.0417	0.0460
–118.2050, 33.9014	0.0523	0.0368#
–118.1272, 34.1326	0.0361	0.0274
–118.0685, 34.0103	0.0457	0.0385
–117.9526, 33.9251	0.0311	0.0628
–117.9385, 33.8306	0.0400	0.0315
–117.8504, 34.1444	0.0280	0.0435
–117.7514, 34.0670	0.0221	0.0532
–117.6291, 34.1037	0.0269	0.0403
–117.4924, 33.9964	0.0496	0.1212
–117.4920, 34.1000	0.1368	0.0617
–117.4160, 33.9996	0.0525	0.0649
–117.2741, 34.1067	0.0274	0.0434
–117.9239, 34.1365	0.0254	0.0568
–118.4564, 34.0511	0.0218	0.0359
–118.4305, 33.9551	0.0575	0.0141#
–118.2200, 33.8025	0.0764	0.1058
**Houston (2 sites)**	**0.0300**	**0.0324**
–95.2576. 29.7337	0.1662	0.0373#
–95.1285, 29.6700	0.0300	0.0324
**Phoenix (3 sites)**	**0.0358**	**0.0256**
–112.1426, 33.4838	0.0395	0.0261
–112.0958, 33.5038	0.0320	0.0251
–112.0466, 33.4580	0.0405	0.0192#
**New York City–Newark (5 sites)**	**0.0283**	**0.0447**
–74.2084, 40.6414	0.0204	0.0536
–74.0663, 40.7316	0.0362	0.0359
–73.8781, 40.8679	0.0704	0.0755#
–73.8215, 40.7361	0.0350	0.0279#
–74.1929, 40.7210	0.0336	0.0462#

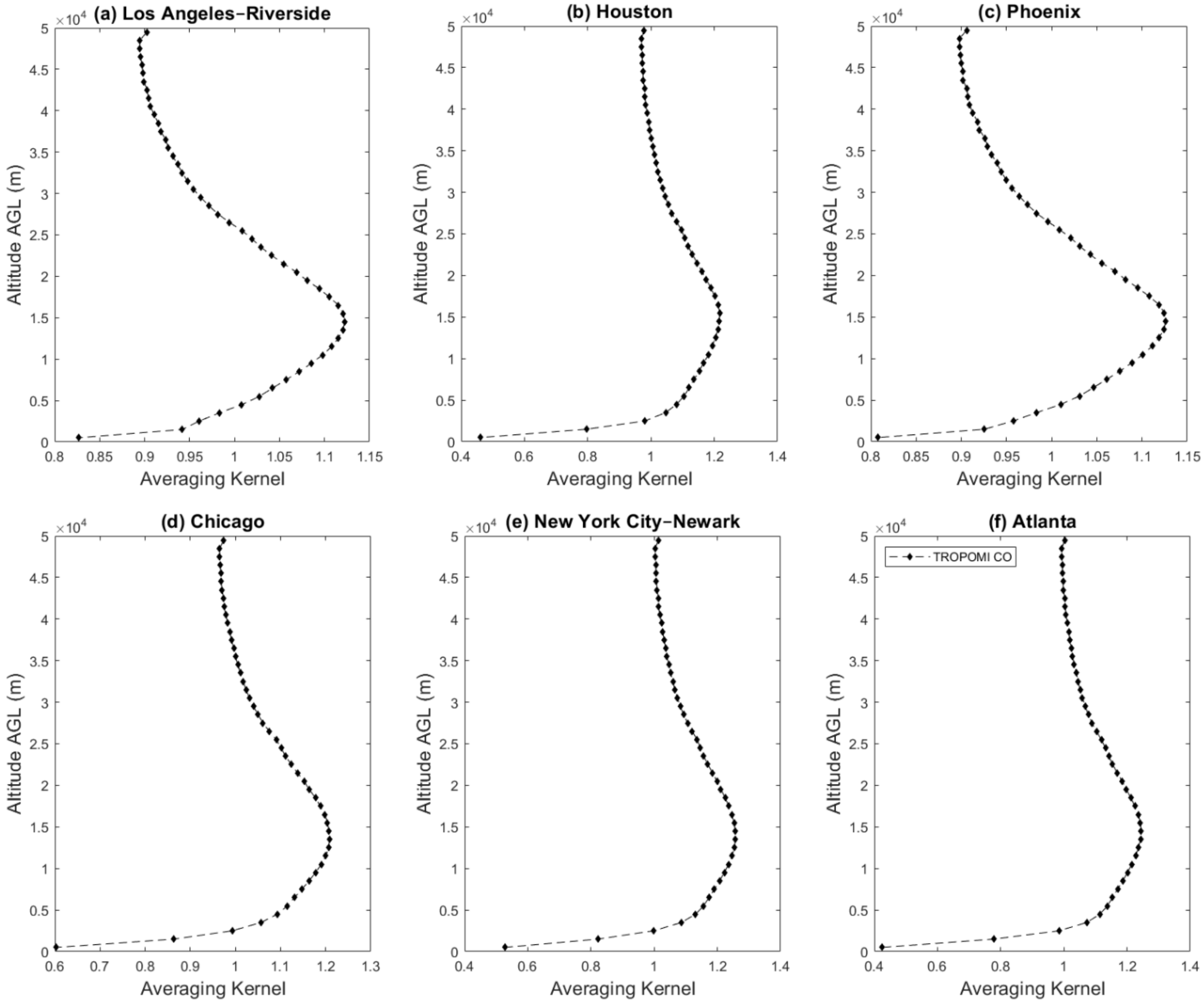

Figure S8. Mean TROPOMI CO average kernel profiles at the reported altitudes above ground level (AGL) for Los Angeles–Riverside (a), Houston (b), Phoenix (c), Chicago (d), New York City–Newark (e), and Atlanta (f) in summer (June–August).

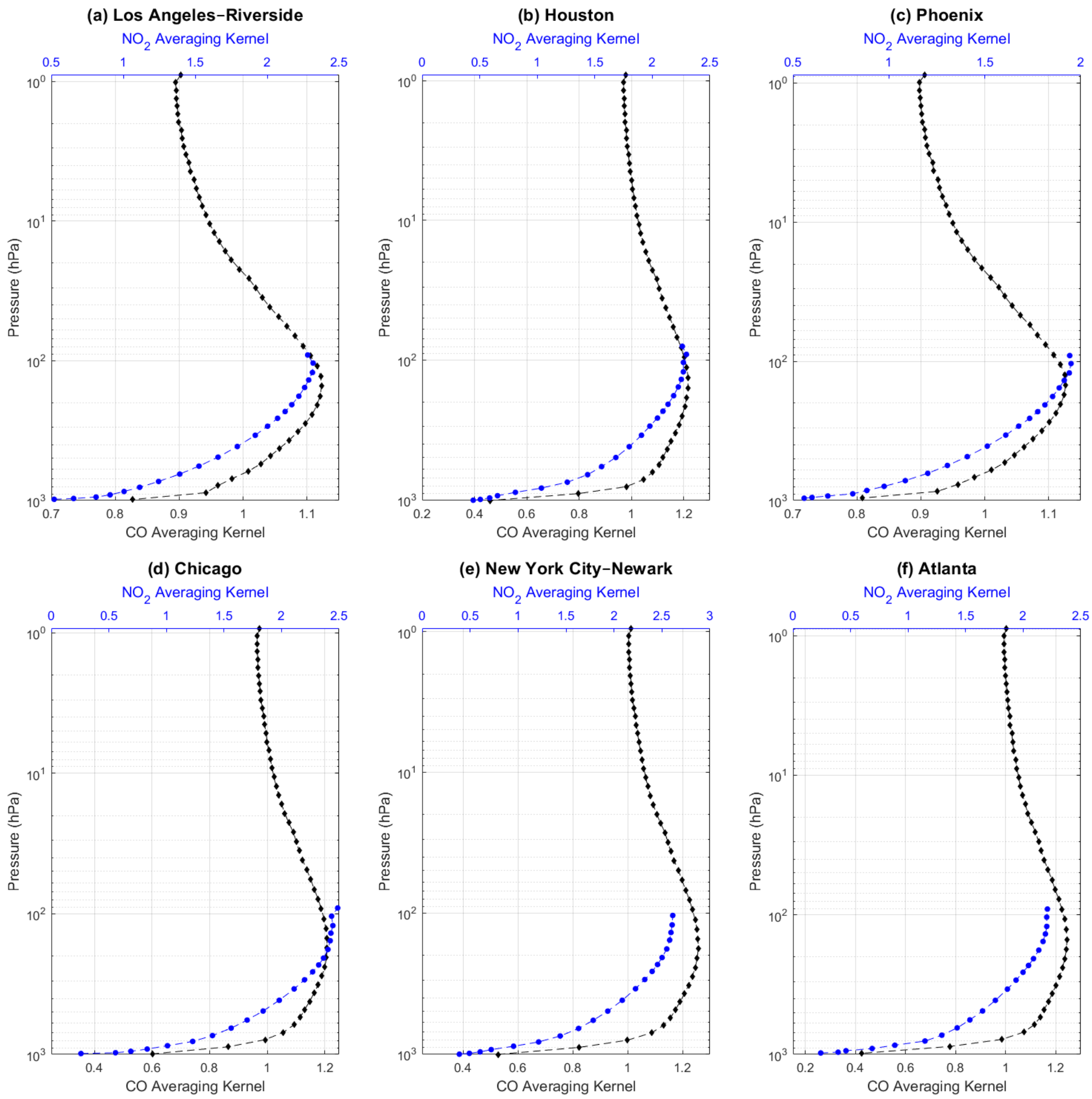

Figure S9. Mean TROPOMI tropospheric NO_2 and full CO averaging kernel profiles at their corresponding pressure levels for Los Angeles–Riverside (a), Houston (b), Phoenix (c), Chicago (d), New York City–Newark (e), and Atlanta (f) in summer (June–August).

References

1. Demetillo, M. A. G.; Harkins, C.; McDonald, B. C.; Chodrow, P. S.; Sun, K.; Pusede, S. E., Space-Based Observational Constraints on NO_2 Air Pollution Inequality From Diesel Traffic in Major US Cities. *Geophysical Research Letters* **2021,** *48* (17), e2021GL094333.

2. Demetillo, M. A. G.; Navarro, A.; Knowles, K. K.; Fields, K. P.; Geddes, J. A.; Nowlan, C. R.; Janz, S. J.; Judd, L. M.; Al-Saadi, J.; Sun, K.; McDonald, B. C.; Diskin, G. S.; Pusede, S. E., Observing Nitrogen Dioxide Air Pollution Inequality Using High-Spatial-Resolution Remote Sensing Measurements in Houston, Texas. *Environmental Science & Technology* **2020,** *54* (16), 9882-9895.

3. Dressel, I. M.; Demetillo, M. A. G.; Judd, L. M.; Janz, S. J.; Fields, K. P.; Sun, K.; Fiore, A. M.; McDonald, B. C.; Pusede, S. E., Daily Satellite Observations of Nitrogen Dioxide Air Pollution Inequality in New York City, New York and Newark, New Jersey: Evaluation and Application. *Environmental Science & Technology* **2022,** *56* (22), 15298-15311.

4. Dressel, I. M.; Zhang, S.; Demetillo, M. A. G.; Yu, S.; Fields, K.; Judd, L. M.; Nowlan, C. R.; Sun, K.; Kotsakis, A.; Turner, A. J.; Pusede, S. E., Neighborhood-level nitrogen dioxide inequalities contribute to surface ozone variability in Houston, Texas. *Environmental Science & Technology–Air* **2024,** *revision*.

5. Sun, K.; Zhu, L.; Cady-Pereira, K.; Chan Miller, C.; Chance, K.; Clarisse, L.; Coheur, P. F.; González Abad, G.; Huang, G.; Liu, X.; Van Damme, M.; Yang, K.; Zondlo, M., A physics-based approach to oversample multi-satellite, multispecies observations to a common grid. *Atmospheric Measurement Techniques* **2018,** *11* (12), 6679-6701.

6. Shindell, D. T.; Faluvegi, G.; Stevenson, D. S.; Krol, M. C.; Emmons, L. K.; Lamarque, J.-F.; Pétron, G.; Dentener, F. J.; Ellingsen, K.; Schultz, M. G.; Wild, O.; Amann, M.; Atherton, C. S.; Bergmann, D. J.; Bey, I.; Butler, T.; Cofala, J.; Collins, W. J.; Derwent, R. G.; Doherty, R. M.; Drevet, J.; Eskes, H. J.; Fiore, A. M.; Gauss, M.; Hauglustaine, D. A.; Horowitz, L. W.; Isaksen, I. S. A.; Lawrence, M. G.; Montanaro, V.; Müller, J.-F.; Pitari, G.; Prather, M. J.; Pyle, J. A.; Rast, S.; Rodriguez, J. M.; Sanderson, M. G.; Savage, N. H.; Strahan, S. E.; Sudo, K.; Szopa, S.; Unger, N.; van Noije, T. P. C.; Zeng, G., Multimodel simulations of carbon monoxide: Comparison with observations and projected near-future changes. *Journal of Geophysical Research: Atmospheres* **2006,** *111* (D19).

7. Winer, A. M.; Peters, J. W.; Smith, J. P.; Pitts, J. N., Jr., Response of commercial chemiluminescent nitric oxide-nitrogen dioxide analyzers to other nitrogen-containing compounds. *Environmental Science & Technology* **1974,** *8* (13), 1118-1121.

8. Dunlea, E. J.; Herndon, S. C.; Nelson, D. D.; Volkamer, R. M.; San Martini, F.; Sheehy, P. M.; Zahniser, M. S.; Shorter, J. H.; Wormhoudt, J. C.; Lamb, B. K.; Allwine, E. J.; Gaffney, J. S.; Marley, N. A.; Grutter, M.; Marquez, C.; Blanco, S.; Cardenas, B.; Retama, A.; Ramos Villegas, C. R.; Kolb, C. E.; Molina, L. T.; Molina, M. J., Evaluation of nitrogen dioxide chemiluminescence monitors in a polluted urban environment. *Atmospheric Chemistry and Physics* **2007,** *7* (10), 2691-2704.

9. Steinbacher, M.; Zellweger, C.; Schwarzenbach, B.; Bugmann, S.; Buchmann, B.; Ordóñez, C.; Prevot, A. S. H.; Hueglin, C., Nitrogen oxide measurements at rural sites in Switzerland: Bias of conventional measurement techniques. *Journal of Geophysical Research: Atmospheres* **2007,** *112* (D11).

10. Russell, A. R.; Valin, L. C.; Bucsela, E. J.; Wenig, M. O.; Cohen, R. C., Space-based Constraints on Spatial and Temporal Patterns of NO_x Emissions in California, 2005−2008. *Environmental Science & Technology* **2010,** *44* (9), 3608-3615.
